# Carpenter bees (Apidae: *Xylocopa*) of Ecuador: distribution, DNA barcodes and plant interactions

**DOI:** 10.7717/peerj.21345

**Published:** 2026-06-16

**Authors:** Raul Ontaneda-Gallegos, Emilia A. Moreno-Coellar, Ana B. García-Ruilova, Fernanda Salazar-Buenaño, Esteban Poveda-Proaño, Daniela Reyes-Barriga, Pamela Lojan-Cueva, Melanie Polo, David A. Donoso, Álvaro Barragán

**Affiliations:** 1 Centro de Investigaciones de la Biodiversidad (CIBIO), Museo QCAZ Invertebrados, Pontificia Universidad Católica del Ecuador, Quito, Ecuador; 2 Grupo de Fisiología del Comportamiento y Sociobiología de Abejas, Instituto de Biodiversidad y Biología Experimental y Aplicada (IBBEA), CONICET–Universidad de Buenos Aires, Buenos Aires, Argentina; 3 Instituto Nacional de Biodiversidad, Quito, Ecuador; 4 Departamento de Biología, Escuela Politécnica Nacional, Quito, Ecuador

**Keywords:** Neotropical bees, Biodiversity, Citizen science, Plant-insect interaction, DNA Barcoding, Biogeography

## Abstract

Knowledge of the diversity and ecology of carpenter bees (*Xylocopa*) in Ecuador remains limited and scattered. Here, we present the first updated assessment of *Xylocopa* across Ecuador, including continental Ecuador and the Galápagos Archipelago, based on the examination of 1,351 specimens from museum collections, published records and citizen science observations. Twenty-two DNA (COI) barcodes were generated for ten species to support accurate identification. In total, 16 *Xylocopa* species were recorded, including three newly reported species for the country: *X. ornata*, *X. metallica*, and *X. nigrocincta*. Six morphotypes could not be confidently assigned to any described species, suggesting the presence of undescribed or cryptic taxa. An identification key to the subgenera and species of *Xylocopa* in Ecuador is also provided. Flower-visitation data was recorded for seven bee species across 60 plant taxa belonging to 26 families. By combining traditional data sources with citizen science observations, this study provides essential baseline information on the diversity, distribution, and ecological interactions of *Xylocopa* in Ecuador. Observed intraspecific phenotypic variation highlights the need for continued morphological and molecular research.

## Introduction

Carpenter bees (genus *Xylocopa* Lepeletier, 1802; Hymenoptera: Apidae) are among the most diverse and ecologically important groups of pollinators. Approximately 500 species have been described, primarily distributed across tropical and subtropical regions worldwide (Michener, 2007). This genus exhibits a pronounced biogeographic split between the Western Hemisphere (America) and the Eastern Hemisphere (Afro-Eurasia and Oceania) (Leys, Cooper & Schwarz, 2002). In the Americas, 15 subgenera are recognized (Melo & Martins, 2025), of which five occur in Ecuador: *Megaxylocopa* Hurd & Moure, 1963, *Neoxylocopa* Michener, 1954, *Notoxylocopa* Hurd, 1956, *Schonnherria* Lepeletier, 1841 and *Xylocopina* Hurd & Moure, 1963 (Zama & Silveira, 2024; Moure & Melo, 2023; Ospina, 2000). However, most of this knowledge comes from scattered museum records and outdated literature, with no recent comprehensive review. The taxonomy of *Xylocopa* in the Neotropics remains challenging due to intraspecific morphological variation, convergent evolution, and the possible existence of undescribed species (Lucia & Gonzalez, 2017; Ramirez-Freire et al., 2012). Consequently, distribution data is often incomplete or unreliable, complicating efforts to assess species conservation status and ecological roles.

*Xylocopa* are distinguished from other bees by their short, sclerotized proboscis with a leaf-shaped galea (Michener, 2007) allowing them to perforate corollas and access concealed nectar (Bernardino & Gaglianone, 2008; Gerling, Velthuis & Hefetz, 1989). They are effective generalist pollinators in both natural and agricultural systems (Keasar, 2010) and frequently visit large, complex flowers in Fabaceae, Malvaceae, Passifloraceae, Myrtaceae, and buzz-pollinated Solanaceae. Their strong thoracic vibrations enable efficient pollen release from poricidal anthers (Jankauski et al., 2022). These interactions contribute to ecosystem resilience and crop productivity, yet remain poorly documented in Ecuador. They are particularly effective pollinators of *Passiflora edulis*, *P. ligularis*, and *Feijoa sellowiana*, often exhibit higher pollination efficiency than *Apis mellifera*, as the smaller honey bees rarely contact the stigma of these large flowers (Barrera, Trinidad & Presas, 2021; Giannini et al., 2020; Gutiérrez-Chacón et al., 2018; Roubik, 1995). Although they are considered generalist pollinators, individual bees may specialize on certain floral types (Araújo et al., 2021), which may explain their sensitivity to habitat disturbance and floral resource loss (Ojija & Adam Silabi, 2024).

Given the morphological complexity and unresolved taxonomy of *Xylocopa*, molecular techniques have become increasingly valuable for accurate species identification. For example, DNA barcoding has proven effective in distinguishing carpenter bees (Agostini et al., 2024; Ruiz et al., 2020), as demonstrated by the successful COI-based identification of *Xylocopa aestuans* in Italy, which showed high similarity to reference sequences from Laos (Flaminio, Bortolotti & Cilia, 2023). However, the accuracy of this approach depends on the quality and geographic representation of reference sequences in global databases, which remains limited in regions such as the Neotropics (Baena-Bejarano et al., 2023). In wild bees, the Barcode of Life Data System (BOLD) facilitates rapid assignment at the genus or species level, but its reliability decreases when validated sequences are lacking, misidentified or incomplete (Smith et al., 2024). Studies that compile information from multiple sources to Ecuadorian bees remain scarce, with only a recent contribution focused on the genus *Bombus* Lepeletier, 1802 (Moreno-Coellar et al., 2025). Therefore, molecular identification should be complemented with detailed morphological examination and comparison against properly identified reference specimens (Honeycutt, 2021).

Here, we present the first updated account of *Xylocopa* in Ecuador, combining distributional records and floral association data, and citizen science observations. Our study aims to (1) provide an updated species list for Ecuador, (2) generate DNA barcodes for 10 species, and (3) document floral associations using citizen science. These data provide a baseline for future ecological, conservation and taxonomical research on this group of pollinators.

## Materials and Methods

### Checklist assembly, identification, and georeferencing

To compile a checklist of *Xylocopa* species from Ecuador, we combined data from museum collections, verified online databases, and published literature. Voucher specimen records were obtained from the Invertebrate Collection of the Pontificia Universidad Católica del Ecuador (QCAZI), the Dry Collection of Invertebrates at the Instituto Nacional de Biodiversidad (INABIO), the Museo de Zoología de la Universidad del Azuay (MZUA), the invertebrate collection of the Museo de Zoología, Universidad Técnica Particular de Loja (MUTPL-CISEC), the Museo de Zoología (LOUNAZ) of the Universidad Nacional de Loja (UNL), and the Natural History Museum Gustavo Orcés V. at the Escuela Politécnica Nacional (MEPN). All records were taxonomically validated through direct examination of specimens. Taxonomic identifications followed the guidelines of the *Catalog of Bees (Hymenoptera, Apoidea) in the Neotropical Region* (Moure, Urban & Melo, 2007). Previously reported distribution records were checked using the Moure Bee Catalogue and the bee species guide available through Discover Life. These resources were used as general references and do not represent exhaustive or fully updated reviews of species distributions. Morphological identification of *Xylocopa* specimens was based on the diagnostic keys of Ferreira et al. (2024b), Mérida-Rivas et al. (2022), Villamizar, Fernández & Vivallo (2020), Mawdsley (2018), and Lucia, Alvarez & Abrahamovich (2014). For species not covered in these works, original species descriptions were consulted. Morphological terminology follows Michener (2007) and Hurd & Moure (1963). Metasomal terga and sterna are indicated as T and S, respectively, and numbered sequentially from anterior to posterior (*e.g*., T1–T7; S1–S6). Ocellar diameter (OD) is used as a relative unit for measurements. Puncture density is expressed in relation to puncture diameter. Antennal flagellar segments are referred to as F, numbered sequentially from the base of the flagellum (*e.g*., F1–F11).

Complementary records were extracted from the Global Biodiversity Information Facility (GBIF; https://www.gbif.org; only museum records), each GBIF record was individually reviewed to verify species identification. Additional data were obtained from the citizen-science platform iNaturalist (https://www.inaturalist.org; only ‘research grade’ records). Citizen-science data provide valuable opportunities to fill long-standing gaps in pollinator research by contributing large volumes of real-time observations that can complement museum and literature records (Callaghan et al., 2020). Such data have proven useful for documenting species distributions, phenology, and ecological interactions across broad geographic scales, particularly in regions lacking systematic monitoring (Chandler et al., 2017; Birkin & Goulson, 2015). Additionally, records from the Barcode of Life Data System (BOLD; https://www.boldsystems.org) were used as a data source. We also reviewed the online photographic collection of the Natural History Museum (London) (https://data.nhm.ac.uk/).

Species delimitation in this study follows an integrative approach, primarily based on morphological diagnosability and supported, when available, by molecular evidence. Molecular data (COI) were used to assess genetic structure and divergence patterns but were not used as the sole criterion for species delimitation.

Records lacking geographic coordinates but containing detailed locality descriptions were georeferenced using cartographic sources, including topographic maps from the Military Geographic Institute and expedition geographic databases. Records with vague locality information (*e.g*., only province or country names) were excluded. Records lacking altitude data were supplemented using Google Earth Online. All verified localities were standardized to the WGS84 coordinate system, and species distribution maps illustrating the extent of occurrence were generated using QGIS (v. 3.44). The complete dataset of verified occurrence records compiled for this study is available in Table S1.

Research activities involving specimen examination and collection were conducted under permits issued by the Ministerio del Ambiente, Agua y Transición Ecológica (MAATE) of Ecuador: permit MAATE-DBI-CM-2024-0440 granted to Pontificia Universidad Católica del Ecuador (PUCE) and permit MAATE-DBI-CM-2023-0309 granted to the Instituto Nacional de Biodiversidad (INABIO).

### Insect–plant interactions and network construction

We compiled records of insect–plant interactions from the citizen science platform iNaturalist (Table S2). We included only photographs showing *Xylocopa* individuals identified to species level and visibly interacting with the flower’s reproductive structures. Plant identifications were validated at the QCA Herbarium, and each species was categorized by its geographical origin (endemic, native, or exotic) following the catalogues of Jørgensen & León-Yánez (1999) and Herrera et al. (2025). In addition, we recorded interactions noted on museum specimen labels (Table S2) that indicated floral visitation. Because these records originate from multiple sources rather than standardized ecological sampling, the dataset reflects heterogeneous sampling effort across regions, plant species, and *Xylocopa* species.

We built interaction networks linking carpenter bees’ records to all the plants identified to species level. For each species, we calculated its degree, defined as the number of interacting species from the opposite trophic level (*i.e*., the number of plant species visited by a carpenter bee or the number of carpenter bee species recorded on a plant). All networks were created in R version 4.3.1 (R Core Team, 2023) using the bipartite package v2.18 (Dormann, Gruber & Fründ, 2008).

### DNA barcoding and tree construction

We processed 23 specimens (Table S3) from the INABIO dry insect collection, the Global Malaise Project (GMECU, Ecuador), the QCAZI, and the Reassembly Project (REASY, Ecuador). Of these, 14 specimens were processed in collaboration with the Canadian Centre for DNA Barcoding (CCDB), and an additional nine specimens were processed at the Biotechnology Laboratory of the Instituto Nacional de Biodiversidad (INABIO).

For the CCDB-processed specimens, DNA was extracted from a single hind leg, amplified, and sequenced for a 568 bp fragment of the mitochondrial cytochrome c oxidase subunit I (COI) gene following standard protocols of the Biodiversity Institute of Ontario, University of Guelph (Wilson, 2012), using the primers C_LepFolF and C_LepFolR.

The nine additional specimens were processed at INABIO using standard molecular procedures. Genomic DNA was extracted from leg tissue preserved in 70% ethanol using the PureLink™ Genomic DNA Mini Kit (K182002; Invitrogen, Waltham, MA, USA), following the manufacturer’s instructions. DNA concentration and quality were assessed prior to PCR amplification. A fragment of the COI gene was amplified using the universal insect primer LCO1490_F (5′-GGTCAACAAATCATAAAGATATTGG-3′) described by Folmer et al. (1994) and the primer LepR1 (5′-TAAACTTCTGGATGTCCAAAAAATCA-3′) described by Hebert et al. (2004). PCR reactions consisted of 4.9 µL of Taq 2X PCR Master Mix (ABM), 0.8 µL of each primer, 2 µL of genomic DNA, 2.4 µL of DNase-free water, and 0.1 µL of BSA. Amplifications were performed in a MiniAmp™ Plus Thermal Cycler (Applied Biosystems) under the following conditions: initial denaturation at 95 °C for 5 min; 35 cycles of 95 °C for 30 s, 48 °C for 30 s, and 72 °C for 50 s; and a final extension at 72 °C for 5 min.

PCR products generated at INABIO were sequenced using Oxford Nanopore Technologies (ONT) on a GridION platform with Flongle flow cells (R10.4.1) and the Rapid Barcoding Kit 96 (SQK-RBK114.96), following the manufacturer’s protocols. Basecalling and demultiplexing were performed in high-accuracy mode using Guppy v6.4.6. Reads with a quality score below 9 were discarded, and consensus sequences were generated using NGSpeciesID (Sahlin, Lim & Prost, 2021).

All COI sequences were submitted for clustering into Barcode Index Numbers (BINs) (Ratnasingham & Hebert, 2013), a provisional taxonomic system that assigns unique identifiers to molecular operational taxonomic units (MOTUs) based on sequence divergence. For clarity, only BINs corresponding to sequences from Ecuadorian specimens are reported within each species catalogue entry (Table S4). BINs from species reported for Ecuador but derived from specimens collected outside the country are also reported in Table S4. Specimen data and sequences are available in BOLD Systems under the dataset “DS-XYLOEC Carpenter Bees (Apidae: *Xylocopa*) of Ecuador” (doi.org/10.5883/DS-XYLOEC). Sequences processed at INABIO were deposited in GenBank (NCBI) and assigned accession numbers (Table S3). BIN assignments reflect the status in BOLD Systems as of February 2026 and may change as new sequences are deposited.

COI sequences were aligned using MAFFT v7 (Katoh & Standley, 2013) under the L-INS-i algorithm with default gap penalties. The reading frame of the alignment was verified by translation using the invertebrate mitochondrial genetic code in MEGA (Tamura, Stecher & Kumar, 2021). Maximum Likelihood (ML) inference was performed in IQ-TREE v3.0.1 (Wong et al., 2025) on the COI alignment partitioned by codon position (Chernomor, Von Haeseler & Minh, 2016). The best substitution models and partitioning scheme were selected using ModelFinder with partition merging (MFP+MERGE) (Kalyaanamoorthy et al., 2017). Node support was assessed with 1,000 ultrafast bootstrap replicates with nearest-neighbor interchange correction (Hoang et al., 2018) and 1,000 SH-like aLRT tests.

One ML tree was generated including only Ecuadorian sequences (Table S3). A second ML tree was generated including Ecuadorian sequences, and sequences from species reported for Ecuador but collected outside the country besides additional reference sequences used to discuss the results (Table S3). The outgroup for these analyses was *Xylocopa* (*Proxylocopa*) *olivieri* Lepeletier, 1841. This choice follows (Melo & Martins, 2025), who demonstrated that subgenus *Proxylocopa* Hedicke, 1938 represents the Old-World sister lineage to all Neotropical *Xylocopa*.

Pairwise Kimura 2-parameter (K2P) (Kimura, 1980) distances were calculated from the 699 bp COI alignment using the ape package v5.7 (Paradis & Schliep, 2019) in R version 4.3.1 (R Core Team, 2023) with pairwise deletion of gaps. The resulting full distance matrix, containing all pairwise comparisons between sequences, is provided in Table S5.

## Results

We curated a total of 1,351 specimens representing 16 described *Xylocopa* species and six morphospecies of uncertain taxonomic status. Of these, 976 specimens representing 18 species were examined from Ecuadorian museum collections. An additional 164 specimen records corresponding to 18 species were obtained from online museum and biodiversity databases. We also incorporated 210 occurrence records representing nine species from the citizen-science platform iNaturalist, as well as four records from three species obtained exclusively from published literature (Table S1). Additional ecological and taxonomic information on Ecuadorian *Xylocopa*, as well as details on species excluded from this checklist, can be found in Article S1.

Due to the absence of an identification key encompassing the subgenera and species from Ecuador, the following keys were developed based on morphological descriptions and diagnostic characters available in the literature (Michener, 2007; Gonzalez, Gonzalez & Cuellar, 2009; Lucia, Alvarez & Abrahamovich, 2014; Mawdsley, 2018; Villamizar, Fernández & Vivallo, 2020; Mérida-Rivas et al., 2022; Ferreira et al., 2024b; Zama & Silveira, 2024), complemented by the examination of specimens deposited in QCAZI and original observations from the present study.

**Genus *Xylocopa* Latreille, 1802**

***Key for Females Subgenera***
**(1)** Mandible tridentate *X*. (*Xylocopina*)
— Mandible bidentate **2**
**(2)** Head with strongly elevated ridges adjacent to ocelli; posterior ocelli above level of eye summits; clypeus distant from eyes (by more than one flagellar diameter) *X*. (*Megaxylocopa*)
— Head without such ridges; posterior ocelli below level of eye summits; clypeus close to eyes **3**
**(3)** Propodeal triangle absent, not delimited by a ridge or line; antennocular distance shorter than interantennal distance *X*. (*Notoxylocopa*)
— Propodeal triangle present and well defined; antennocular distance equal to or greater than interantennal distance **4**
**(4)** Metasomal sterna with distinct longitudinal ventral carina; clypeus delimited by a continuous smooth ridge along the epistomal suture; integument dull *X*. (*Neoxylocopa*)
— Metasomal sterna without distinct ventral carina; clypeus without a continuous ridge; integument usually with a faint metallic sheen *X*. (*Schonnherria*)

***Key for Males Subgenera***
**(1)** Eyes often enlarged and convergent above **2**
— Eyes small and not convergent above **3**
**(2)** T7 without posterolateral projections, integument usually metallic sheen *X*. (*Schonnherria*)
— T7 with posterolateral projections, integument without metallic sheen *X*. (*Notoxylocopa*)
**(3)** Integument and pubescence predominantly ferruginous/yellowish, clypeus and face always predominantly light-colored **4**
— Integument predominantly black, clypeus yellowish only on the sides with a black central band; upper lateral margin of clypeus abruptly and steeply elevated *X*. (*Xylocopina*)
**(4)** Inner apical margin of the hind tibia with rounded apex, ventral surface of posterior tibia with a strong declivity, juxtantennal carina absent *X*. (*Megaxylocopa*)
— Hind tibia, internal distal extreme with blunt or curved projection, juxtantennal carina present, though sometimes only weakly indicated *X*. (*Neoxylocopa*)

**Subgenus *Megaxylocopa* *Hurd & Moure, 1963***

***Key for Subgenus Megaxylocopa Females***
**(1)** Frons with a broad transverse carina ending laterally in horn-like projections near the lateral ocelli; median ocellus below the level of the lateral ocelli and of the upper eye margins; carina lateral end close to the eye (≈2× the diameter of the median ocellus); T1–T3 with sparse minute punctures *X. fimbriata*
— Frons with a narrow arc-shaped carina or pair of curved elevations beside the median ocellus; median ocellus at or slightly below the level of the lateral ocelli and near or above the upper eye margins; carina lateral end far from the eye (>4× the diameter of the median ocellus); T1–T3 with dense minute punctures *X. frontalis*

***Key for Subgenus Megaxylocopa Males***
**(1)** Terga yellowish-orange without apical bands; metasoma entirely covered with yellowish-orange pubescence *X. fimbriata*
— Terga yellow with broad black apical bands; T7 yellow; punctuation sparse between parapsidal line and discal area of scutum *X. frontalis*

***Xylocopa* (*Megaxylocopa*) *fimbriata* Fabricius, 1804**

In Ecuador, *Xylocopa fimbriata* is known from a single historical specimen at the United States National Museum, Smithsonian Institution (USNM; USNMENT01733603), labeled simply as collected in Ecuador and noted by Hurd (1978); its identification was confirmed *via* museum photos. The species occurs across Central and South America and the Caribbean (Moure & Melo, 2023). Although some reports list this species as occurring in Bolivia and Peru, these records are likely erroneous. The confusion may stem either from misidentified specimens or from incorrect locality information, as there is no verifiable evidence supporting its presence in either country (Mérida-Rivas et al., 2022). Despite the review of major institutional collections, no additional specimens have been documented from Ecuador. This absence of recent records suggests that the species may be locally rare or currently undetected in the country. Females nest in seasoned wood and exhibit aggressive interactions while males establish hovering territories (Janzen, 1966), potentially competing for resources with other *Xylocopa* species. Given the scarcity of records and its uncertain current presence, *X. fimbriata* should be considered a species of potential conservation concern in Ecuador. Due to the lack of precise locality data, we did not generate a distribution map. Females are distinguished by a wide frontal carina with lateral ends near the eyes forming horn-like prominences between the middle and lateral ocelli when viewed laterally. Males are characterized by yellowish-orange metasomal terga without bands (Mérida-Rivas et al., 2022; Gonzalez, Gonzalez & Cuellar, 2009).

***Xylocopa* (*Megaxylocopa*) *frontalis* Olivier, 1789**

**Figure 1:**
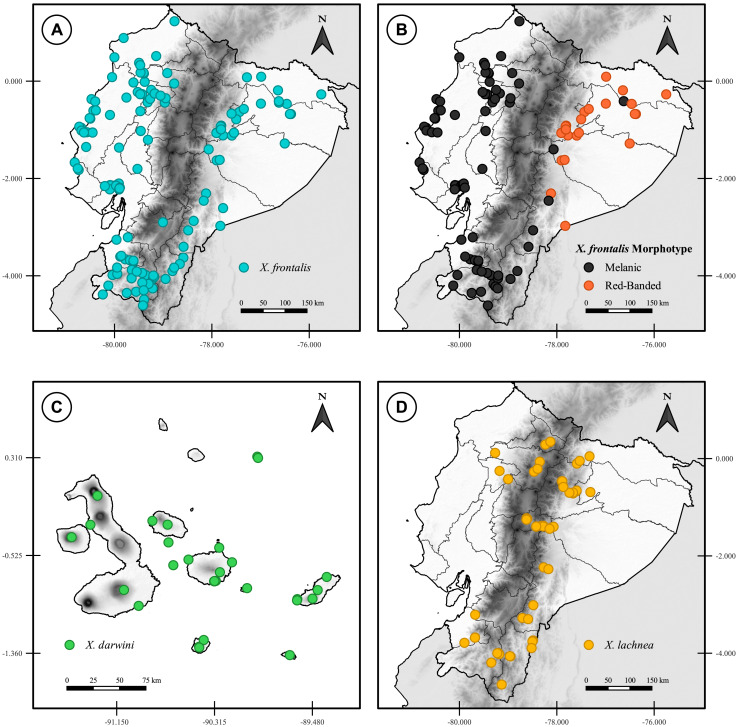
Distribution records. (A) *Xylocopa* (*Megaxylocopa*) *frontalis*; (B) Distribution of metasomal color morphs of *X*. (*M*.) *frontalis*; (C) *Xylocopa* (*Neoxylocopa*) *darwini*, Galápagos archipelago; (D) *Xylocopa* (*Neoxylocopa*) *lachnea*.

*Xylocopa frontalis* is a widely distributed carpenter bee ranging from Mexico to Argentina (Moure & Melo, 2023). In Ecuador, it occurs from sea level up to 2,548 m a.s.l. (Fig. 1A), although the highest record may reflect a generalized locality assigned to the nearest major city. The next confirmed record at 2,332 m a.s.l. extends the known altitudinal range for the species, previously reported as 0–2,180 m a.s.l. by Mérida-Rivas et al. (2022). Some individuals exhibit terga coloration. This trait is sometimes used in *Xylocopa* species diagnoses, although its taxonomic reliability for this species within the country is limited due to intraspecific variation. A morphotype with entirely black terga is found in the coastal region, southern highlands (Loja), and southern Amazon (Zamora Chinchipe and Morona Santiago), while another morph with red bands on the terga occurs in the central and northern Amazon. A few black individuals have also been recorded in the northern Amazon (Sucumbíos), indicating that the two morphs are largely segregated but likely come into contact (Fig. 1B). Males are characterized by a middle ocellus smaller than the lateral ocelli, ventral surface of the hind tibia with a strong declivity, and metasomal terga with finer and sparser punctuation. Males examined from both the eastern and western slopes of the Andes consistently showed the inner apical projection of the hind tibia as a single rounded projection, lacking the broad anterior lobe described by Mérida-Rivas et al. (2022). Only a slight indentation on the anterior margin was observed in its place. Females are distinguished by a narrow subocellar carina with lateral ends positioned far from the eyes and juxtantennal carina absent (Ferreira et al., 2024b; Mérida-Rivas et al., 2022; Gonzalez, Gonzalez & Cuellar, 2009).

**DNA barcode.** The COI sequence (658 bp) for the melanic morphotype was assigned the BIN: AGK0908. This BIN appears to be unique to Ecuador (Table S4).

**Subgenus *Neoxylocopa* Michener, 1954**

***Key for Subgenus Neoxylocopa Females***
**(1)** Mesosoma entirely covered with orange or reddish-orange pubescence; T1 sometimes completely, sometimes partially covered with orange or reddish-orange pubescence; tegulae reddish; disc of T2–T5 sparsely covered with very short black hairs *X. similis*
— Mesosomal pubescence entirely black; tegulae black **2**
**(2)** Tergal pubescence dense and plumose, hairs long and concealing the integument; wings dark brown to black with violet reflections *X. lachnea*
— Tergal pubescence short and sparse, integument clearly visible **3**
**(3)** Metasoma with reddish bands *X. nigrocincta*
— Metasoma entirely black **4**
**(4)** Gena with fine, scattered punctures separated by >3× puncture diameter; integument smooth and shining between punctures *X*. sp. 2
— Gena moderately punctate, punctures separated by 1–2**×** their diameter, giving the surface a somewhat coarse appearance **5**
**(5)** Scutellum convex, dorsal surface rounded, lacking a carinate edge at the transition to the posterior surface (frontal view); wings smoky with strong violet reflection (Galápagos) *X. darwini*
— Scutellum slightly convex, with an abrupt transition to the posterior surface forming a weakly carinate edge (frontal view); wings usually lighter, coppery with greenish to bluish reflections (continent) *X*. sp. 1

***Key for Subgenus Neoxylocopa Males***
**(1)** T2–T7 with long pubescence, obscuring the integument, which is barely visible; hind tibia with a long, robust external spur *X. lachnea*
— Only T6–T7 with long pubescence, integument of T2–T5 clearly visible; hind tibia with a short, slender external spur **2**
**(2)** Metasomal terga orange, with broad black apical bands covering nearly half of each tergum; hind tibia distal subapical projection globose *X. similis*
— Metasomal terga yellow, with black apical bands; hind tibia distal subapical projection robust **3**
**(3)** Found on the Galápagos Archipelago *X. darwini*
—Found on the Pacific coast of continental Ecuador *X*. sp. 1

*Males of *X. nigrocincta* were not examined

***Xylocopa* (*Neoxylocopa*) *darwini* Cockerell, 1926**

*Xylocopa darwini* is the only carpenter bee endemic to Ecuador and is confined to the Galápagos Archipelago. It occurs on Isla Santiago, Isla Genovesa, Isla Santa Cruz, Isla Pinzón, Isla Rabida, Isla Santa Fe, Isla Española, Isla San Cristóbal, Isla Floreana, Isla Plaza Sur, Isla El Edén, Isla Seymour Sur, Isla Isabela, and Isla Fernandina (Fig. 1C). This species is difficult to identify due to its close similarity to *Xylocopa* sp. 1. The female wings are smoky with a strong violet reflection, a greenish reflection at the base, and occasionally a blue reflection. This species can be differentiated from the other completely melanic species of the *Neoxylocopa transitoria* group present in Ecuador by its convex scutellum, which has a rounded dorsal surface lacking a carinate edge at the transition to the posterior surface when viewed frontally. The male has a clypeus bearing a broad black longitudinal line; terga yellowish-orange to brown, with black integumental bands along the posterior margins with variable width among individuals; metasoma with yellow pubescence. Hind tibia with a prominent subapical projection on the internal distal extreme, robust and slightly curved distally; basal portion of this process bearing a narrow carinate margin running diagonally from the apical section and extending along nearly one third of the internal posterior margin of the tibia.

**DNA barcode.** The first COI sequence (658 bp) for *X. darwini* was assigned BIN AFA9827 (Table S4) for specimens from Isla Santa Cruz and Isla Isabela, while the second sequence (658 bp) was assigned BIN AFE8750 (Table S4). The latter was found exclusive to Isla Isabela.

***Xylocopa* (*Neoxylocopa*) *lachnea* Moure, 1951**

It has been recorded from Colombia, Ecuador, and Peru (Moure & Melo, 2023). In Ecuador, *X. lachnea* is common on the eastern slopes of the Andes and in inter-Andean valleys, with fewer records from the western slope, at elevations between 143 and 2,675 m a.s.l. (Fig. 1D). Both males and females are characterized by dense, long, plumose pubescence on the metasomal terga, sufficiently dense to obscure the underlying integument (Gonzalez, Gonzalez & Cuellar, 2009). The male’s hind tibia internal distal subapical projection is weakly developed, nearly obsolete, flattened. A prominent tibial spur is noticeable at the distal extreme. *X. lachnea* forages mainly in the forest canopy and is an important pollinator of Passifloraceae (Pinilla-Gallego & Nates-Parra, 2015).

***Xylocopa* (*Neoxylocopa*) *nigrocincta* Smith, 1854**

Synonym: *Xylocopa* (*Neoxylocopa*) *suspecta* Moure & Camargo, 1988

**Figure 2:**
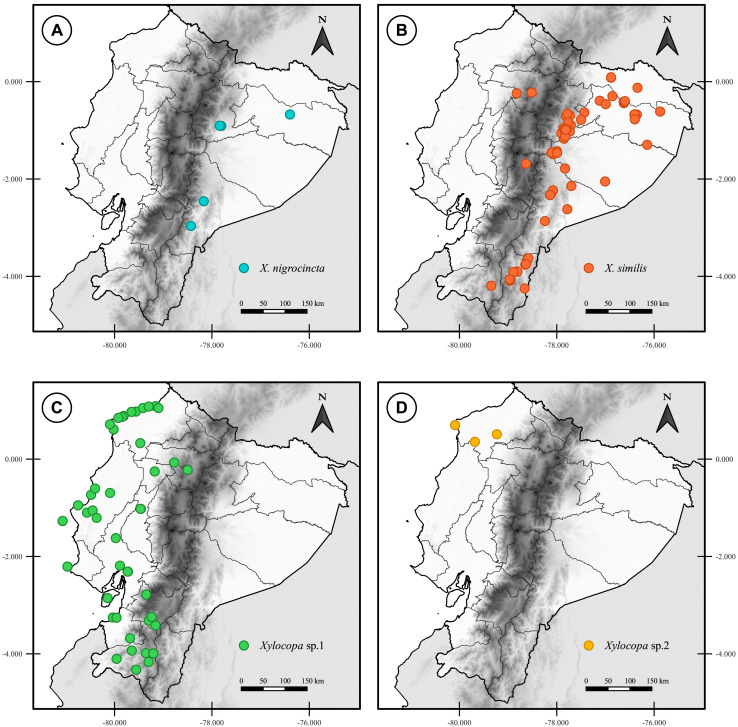
Distribution records. (A) *Xylocopa* (*Neoxylocopa*) *nigrocincta;* (B) *Xylocopa* (*Neoxylocopa*) *similis;* (C) *Xylocopa* (*Neoxylocopa*) sp. 1; (D) *Xylocopa* (*Neoxylocopa*) sp. 2.

Moure’s catalog lists *X. nigrocincta* from Argentina, Brazil, and Paraguay, and *X. suspecta* from Argentina, Bolivia, Brazil, Paraguay, and Uruguay (Moure & Melo, 2023). This study records *X. nigrocincta* for the first time in Ecuador. It occurs throughout the Amazonian region at elevations of 218 to 1,046 m a.s.l. (Fig. 2A). The recent synonymy with *X. suspecta* showed that this species exhibits variation in metasomal coloration. Specimens from the northwestern Brazilian Amazon are entirely black, while individuals from northern Argentina and southern Brazil show colored terga (Agostini et al., 2024). Ecuadorian specimens resemble the southern morphotype, with red to yellowish bands on T1–T4. These markings have led to misidentifications with *X*. (*Megaxylocopa*) *frontalis*, which has similar red metasomal bands. Such confusion may also occur in citizen science records on platforms like iNaturalist. The coexistence of the colored morphotype of both species in the same region suggests possible convergence in coloration or environmental influences on pigment expression. Ecuadorian specimens have been reported visiting flowers of Bixaceae. Females are recognized by the absence of ocellar carina and red banded terga (Ferreira et al., 2024b; Lucia, Alvarez & Abrahamovich, 2014). The presence of these bands is still uncertain as a diagnostic trait in Ecuador. The male was not found in any of the examined collections and therefore not studied here, its description is available in Lucia, Alvarez & Abrahamovich (2014).

***Xylocopa* (*Neoxylocopa*) *similis* Smith, 1874**

Moure & Melo (2023) report *X. similis* in Brazil, Colombia, Ecuador, and Peru. In Ecuador, it occurs on the eastern slopes and in the lowland rainforests across all Amazonian provinces and in Loja at 204–1,685 m a.s.l. (Fig. 2B). We found a few unusual records from Riobamba at 2,736 m a.s.l. and Quito at 2,814 m a.s.l., both labeled with the names of the major cities and located over 1,000 m above the next highest known occurrences. An additional unusual record from Santo Domingo de los Tsáchilas at 1,945 m a.s.l. represents the only known occurrence of the species on the western slopes of the Andes. Females of this species are recognized by reddish hairs on the mesosoma and orange pubescence on the first metasomal terga (Mawdsley, 2018; Gonzalez, Gonzalez & Cuellar, 2009). Males show a clypeus with an orange central patch and a thin longitudinal line of variable length within individuals. The mesosoma and metasoma are predominantly reddish-brown with orange pubescence. The terga have wide black integumental bands along the posterior margins. The hind tibia distal subapical projection is rounded, inflated, and somewhat globose, and its posterior margin forms a narrow laminar ridge that extends along approximately half of the tibial length.

***Xylocopa* (*Neoxylocopa*)** **sp. 1**

This species occurs along the Ecuadorian coast and the eastern slopes of the Andes, including Isla de la Plata and the southern lowland Andean areas, from sea level up to 2,059 m a.s.l. (Fig. 2C). Two additional records, one from Quito at 2,901 m a.s.l. and another from Azuay at 2,726 m a.s.l., are notably higher than the next confirmed record and may represent isolated occurrences or imprecise locality data. Original identification as *Xylocopa* (*Neoxylocopa*) *transitoria* Pérez, 1901 was based on the syntype determined by Dr. Suzanne Batra at QCAZI. A later comparison with the key of Ferreira et al. (2024b) revealed that the Ecuadorian specimens differ from the diagnostic characters listed for *X. transitoria*, in two main features: the scutellum is dorsally flat to slightly convex, with an abrupt transition to the posterior surface that forms a weakly carinate edge, rather than the concave dorsal surface and strong carinate edge described for *X. transitoria*. In addition, while *X. transitoria* was described as having dark bluish-black wings with blue-violet reflections (Pérez, 1901), Ecuadorian specimens typically exhibit lighter coppery wings with greenish or bluish reflections, with violet reflections being rare, occurring only in few individuals. The scutellar morphology observed here is shared with other *Neoxylocopa* species, such as *X. orthogonaspis* Moure, 2003. Males of this species were indistinguishable from males of *X. darwini* in all diagnostic traits, including the hind tibial process. Of the historical material reported by Cockerell (1914), only two females remain confidently identified as *X. transitoria*. Two additional specimens from the same series appear to have undergone subsequent reidentification or labeling changes and are now either assigned to the genus level.

**DNA barcode.** The COI sequence (681 bp) was assigned to BIN AHI7092 (Table S4). No identical sequences were found in the BOLD BIN database; however, the nearest neighbor corresponded to BIN AFA9827 (Table S4), associated with *X. darwini*.

***Xylocopa* (*Neoxylocopa*)** **sp. 2**

Known from Esmeraldas province between 129 and 522 m a.s.l. (Fig. 2D). The female of this species can be recognized by its entirely black integument and pubescence; wings light brown with faint greenish iridescence across the entire surface and black venation. The gena with fine, scattered punctures separated by more than three puncture diameters, the integument smooth and shining between punctures, and the vertex finely punctate. These characters suggest a possible affinity with *Xylocopa (Neoxylocopa) columbiensis* Pérez, 1901, described from Panama, which shares sparsely punctate gena and supraocular areas, similar tergal puncturation, wing coloration, and scutellum shape (Lucia & Gonzalez, 2017). No male specimens were found during this study.

**DNA barcode**. The COI sequence (658 bp) was assigned to BIN AGB1358 (Table S4). No identical sequences were found in the BOLD BIN database, leaving the species identification uncertain.

**Subgenus *Notoxylocopa* Hurd, 1956**

***Xylocopa* (*Notoxylocopa*) *tabaniformis* Smith, 1854**

This species is distributed in Colombia, Costa Rica, Ecuador, Guatemala, Mexico, and Panama (Moure & Melo, 2023). It is the only representative of the subgenus *Notoxylocopa* reported from Ecuador. Females are distinguished by the absence of a propodeal triangle, while males exhibit T7 with a bidentate margin (Michener, 2007; Hurd & Moure, 1963). Two specimens from Ecuador are recorded in the USNM collection, both originating from the Charles Fuller Baker collection. These specimens appear to correspond to the Ecuadorian material cited by O’Brien & Hurd (1965). However, one male reported by those authors from the same provenance could not be located. No additional material was found in national collections or during recent surveys. We believe these specimens could have been retrieved by Robert Asa Cushman in 1927 from the Philippines and later transferred to the Smithsonian Institution (Cushman, 1928). There are no records that Baker ever visited Ecuador. His documented work in South America includes the H. H. Smith expedition to the Santa Marta Mountains (1898–1899) and his time as Curator at the Museu Goeldi in Pará, Brazil (1907–1908) (Charles Fuller Baker, 1927). The Ecuadorian label on these specimens may be erroneous or the result of imprecise historical labeling, especially considering the territorial disputes and undefined borders in the Amazon region of Ecuador at that time. The specimens of *X*. (*Schonnherria) muscaria* and *X*. (*Megaxylocopa*) *fimbriata* come from the same collection, suggesting a shared provenance and uncertainty about their true origin.

**Subgenus *Schonnherria* Lepeletier, 1841**

***Key for Subgenus Schonnherria Females***
**(1)** Labrum with a single large capitate tubercle about the size of the median ocellus **2**
— Labrum with three tubercles, or a median elongated tubercle and two smaller sublateral ones **3**
**(2)** T3–T6 with orange pubescence hairs *X. metallica*
— Metasomal terga with black pubescence with whitish hairs on T4 *X*. sp. 4
**(3)** Mid and hind tibia and basitarsi with whitish or yellowish hairs **4**
— Mid and hind tibia and basitarsi with black hairs **7**
**(4)** Gena and vertex finely punctate, integument with metallic blue-green sheen; T1–T6 with orange pubescence *X. ornata*
— Gena and vertex coarsely punctate; T1–T6 without orange pubescence **5**
**(5)** Mesosoma with pale yellowish-brown hairs; integument black with greenish tints; T2–T4 with white apical bands *X. viridis*
— Mesosoma and metasoma with black pubescence, bluish-violet sheen; whitish hairs only on hind tibia and basitarsi **6**
**(6)** Hind basitarsi setation cream on the outer side and black on the inner side *X. ecuadorica*
— Hind basitarsi setation cream on the outer side and red on the inner side *X. piurensis*
**(7)** All pubescence black throughout **8**
— Paraocular area and frons with white hairs; metasoma with white hairs laterally on the terga **9**
**(8)** Integument black with inconspicuous greenish reflections; large body size (body length ~22 mm) *X. viridigastra*
— Integument black with conspicuous bluish highlights on the head and mesosoma and greenish reflections on the metasoma; small body size (body length ~15 mm) *X*. sp. 3
**(9)** Gena densely covered by white plumose hair *X. muscaria*
— Only lower gena covered in white plumose hairs; upper gena glabrous with scattered black hairs *X. lucida*

***Key for Subgenus Schonnherria Males***
**(1)** Clypeus integument black with metallic highlights *X*. sp. 5
— Clypeus integument light predominantly yellow without metallic sheen **2**
**(2)** Clypeus, supraclypeal area, and labrum glabrous; scape entirely black; yellow markings on head restricted to the labrum, clypeus, and supraclypeal area; paraocular area dark *X. muscaria*
— Clypeus, supraclypeal area, and labrum with plumose hairs; yellow markings present on the scape and extending across the clypeus, supraclypeal area, and lower paraocular area **3**
**(3)** Middle and hind legs with predominantly black pubescence on the outer surface **4**
— Outer surface of middle and hind legs with yellow or ferruginous pubescence **5**
**(4)** Integument nearly black with an inconspicuous green metallic sheen; pubescence grayish on T1 and on the anterior and underside of the mesosoma, entirely black elsewhere *X. viridigastra*
— Integument black with conspicuous bluish highlights on the head and mesosoma and greenish reflections on the metasoma; pubescence completely black on mesosoma *X*. sp. 3
**(5)** Compound eyes strongly convergent dorsally (<3× OD); yellowish hairs on the clypeus and paraocular area; F1 dark; white pubescent bands on metasomal terga absent *X. ornata*
— Compound eyes slightly convergent dorsally (>3× OD); dark hairs on the clypeus and paraocular area; F1 with a yellow marking; white pubescent bands present along the distal margins of T2–T5 **6**
**(6)** Tegula reddish brown; pubescence predominantly yellowish to whitish; mesosoma with pale yellowish-brown hairs; terga with coarse and dense punctures (1–2× puncture width) *X. viridis*
— Tegula black; pubescence mostly black, with some grayish hairs on the metasoma; terga with dense punctures (0.5–1× puncture width) *X. ecuadorica*

*Males of *X. metallica*, *X. piurensis*, and *X. lucida* were not examined and remain undescribed

***Xylocopa* (*Schonnherria*) *ecuadorica* Cockerell, 1909**

**Figure 3:**
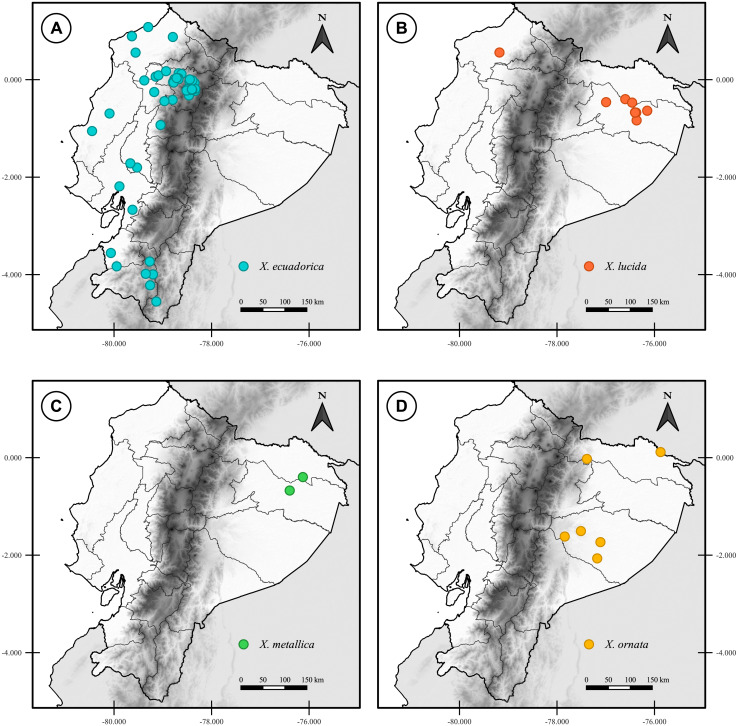
Distribution records. (A) *Xylocopa* (*Schonnherria*) *ecuadorica;* (B) *Xylocopa* (*Schonnherria*) *lucida;* (C) *Xylocopa* (*Schonnherria*) *metallica*; (D) *Xylocopa* (*Schonnherria*) *ornata*.

*Xylocopa ecuadorica* occurs in the Neotropical region, with confirmed records in Colombia, Ecuador, and Panama (Moure & Melo, 2023). In Ecuador, it is found in the Coastal region, the Inter-Andean valleys, and the Amazonian region, from sea level up to 2,814 m a.s.l. (Fig. 3A). In Colombia, it has only been reported from the Pacific coast (Chocó) (Villamizar, Fernández & Vivallo, 2020). Historically treated as a subspecies of *Xylocopa varians* Smith, 1874, it is now recognized as a distinct species following Moure, Urban & Melo (2007) and molecular evidence (Melo & Martins, 2025). This species exhibits metallic bluish and violet highlights on the head, bluish reflections on the mesosoma, and greenish metallic highlights on the metasoma. Traits previously proposed by Cockerell (1912) to distinguish species in the former *varians* group, such as outer hind basitarsus hair coloration, show considerable variation among individuals across Ecuador, ranging from pale or whitish to reddish tones, making identification based solely on these characteristics unreliable. Given the overlap of diagnostic traits and the presence of both *X. ecuadorica* and *X. piurensis* in Ecuador, a comprehensive taxonomic revision, incorporating morphological and molecular data, is needed to determine more diagnosis traits for these species. Males examined in this study show mostly black pubescence on the mesosoma, sometimes appearing slightly gray, differing from the yellowish pale pubescence reported by Villamizar, Fernández & Vivallo (2020).

**DNA barcode.** The COI sequence (658 bp) was assigned to BIN AET4851. This BIN is newly associated with *X. ecuadorica* and includes records from Ecuador and Costa Rica (Table S4). *X. ecuadorica* has not been reported from Costa Rica in the distributions summarized by Moure & Melo (2023) or Ascher & Pickering (2020). Although specimens identified as *X. ecuadorica* from Costa Rica are listed in GBIF, these records have not been critically evaluated yet. BIN data suggest genetic consistency with *X. ecuadorica*, indicating a potentially broader distribution, pending confirmation through morphological examination of Costa Rican specimens.

***Xylocopa* (*Schonnherria*) *lucida* Smith, 1874**

In Ecuador, *X. lucida* has been observed in the Amazonian provinces of Orellana and Sucumbíos, at a narrow elevation range between 164 and 253 m a.s.l. (Fig. 3B). Only one specimen was found on the western slopes of the Andes, where it has been reported in Colombia (Villamizar, Fernández & Vivallo, 2020); however, this record should be considered tentative pending a formal description and confirmation of the male. Although this species is listed by Ascher & Pickering (2020) for Ecuador, it is not included in Moure’s catalogue (2023), which only reports it for neighboring countries. *X. lucida* is often misidentified as *X. muscaria* due to their similar coloration, but can be distinguished by its glabrous upper gena with scattered whitish hairs below, in contrast to the dense whitish plumose pubescence of *X. muscaria*.

**DNA barcode.** The COI sequence (658 bp) was assigned to BIN AFQ4985 (Table S4). The specimen housed at TUM (Technische Universität München) is a male, representing the first known male of *X. lucida* and the only record of the species from Ecuador on the western slopes of the Andes. This BIN also includes two female specimens identified based on photographs, including a public record from the Peruvian Amazon in the Madre de Dios Department (Table S4). Although the molecular evidence supports the identification of the Ecuadorian specimen as *X. lucida*, a formal description of this male specimen is needed to establish diagnostic characters for the species.

***Xylocopa* (*Schonnherria*) *metallica* Smith, 1874**

*Xylocopa metallica* has been recorded in Brazil, Colombia, Peru, Suriname, and Guyana (Moure & Melo, 2023; Ascher & Pickering, 2020; Mawdsley, 2018). Our study documents the first records of *X. metallica* in Ecuador, represented by two individuals collected at 219 m a.s.l. in Sucumbíos and 218 m a.s.l. in Orellana, both within the Amazonian region (Fig. 3C). Females are recognized by a large capitate tubercle at the basal area of the labrum, dark pubescence on T1–T2, and yellowish to orange pubescence on T4–T6 (Villamizar, Fernández & Vivallo, 2020; Mawdsley, 2018). The male of *X. metallica* remains undescribed.

***Xylocopa* (*Schonnherria*) *muscaria* Fabricius, 1775**

This species is widespread in Central and South America, including Ecuador (Moure & Melo, 2023). *X. muscaria* is reported to be among the most frequently collected species of the subgenus *Schonnherria* (Villamizar, Fernández & Vivallo, 2020). In Ecuador, however, it is known from only a few verified specimens housed at the USNM, collected by Charles Fuller Baker and labeled only to country level, with no date information available. Deforestation, agricultural expansion, and forest fragmentation in the Ecuadorian Amazon may contribute to the contraction of its range into isolated habitat patches (Jones et al., 2023; López, 2022). Studies across the Neotropics show that formerly abundant insect species are declining disproportionately (Van Klink et al., 2024), and *X. muscaria* may be following the same pattern within the country. No recent or localized records are known from Ecuador, and its ecological habits in the country remain unstudied. Because of the lack of precise locality data, no distribution map was generated for this species.

***Xylocopa* (*Schonnherria*) *ornata* Smith, 1874**

*Xylocopa ornata* is distributed across Bolivia, Brazil, Colombia, Peru, and Venezuela (Moure & Melo, 2023; Ascher & Pickering, 2020; Mawdsley, 2018). We report the first records of this species in Ecuador. It can be found in the lowland Amazon between 223 and 942 m a.s.l. in the Pastaza and Sucumbíos provinces (Fig. 3D). Females are recognized by strong metallic blue and green highlights on the body and orange to yellow pubescence on T1–T6 (Villamizar, Fernández & Vivallo, 2020; Mawdsley, 2018). No male specimens were encountered during this study; the original description of the male can be found in Zama & Silveira (2024).

**DNA barcode.** The COI sequence (658 bp) for the Ecuadorian specimen of *X. ornata* was assigned BIN: AGL3951 (Table S4). No other individuals currently share this BIN.

***Xylocopa* (*Schonnherria*) *piurensis* *Cockerell, 1912***

**Figure 4:**
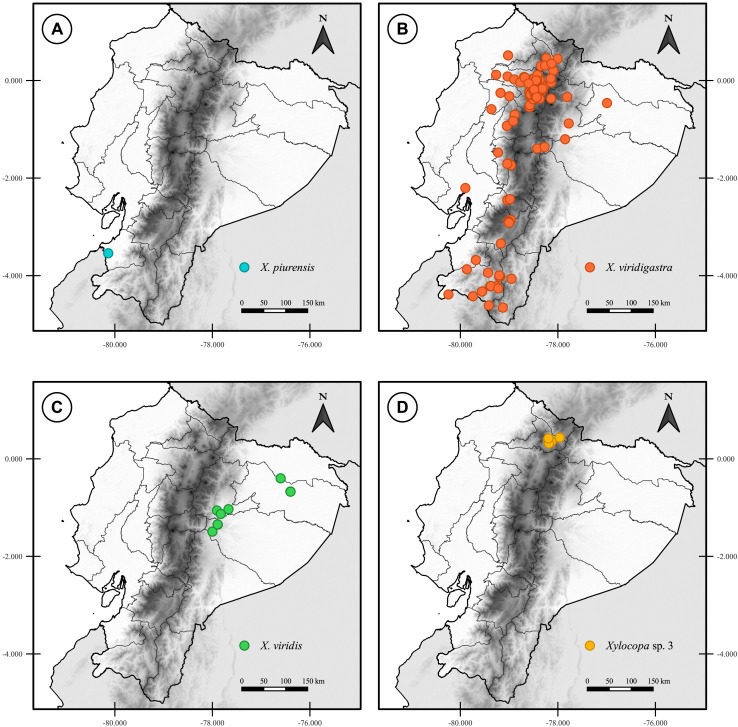
Distribution records. (A) *Xylocopa* (*Schonnherria*) *piurensis;* (B) *Xylocopa* (*Schonnherria*) *viridigastra;* (C) *Xylocopa* (*Schonnherria*) *viridis*; (D) *Xylocopa* (*Schonnherria*) sp. 3.

*Xylocopa piurensis* occurs along the coasts of northern Peru and southern Ecuador in seasonally dry forests and coastal desert habitats from sea level up to 1,500 m (Moure & Melo, 2023; Juárez-Noé & González-Coronado, 2018). Identification of *X. piurensis*, *X. ecuadorica*, and *X. incarum* Cockerell, 1911 relies primarily on hind basitarsi hair coloration: *X. incarum* is entirely red, *X. piurensis* is cream on the outer surface and red on the inner surface, and *X. ecuadorica* is cream on the outer surface and black on the inner surface (Cockerell, 1912). Both *X. piurensis* (Fig. 4A) and *X. ecuadorica* (Fig. 3A) were found in the same habitat in Ecuador (Arenillas, El Oro). The close geographic overlap of these species in southern Ecuador and northern Peru complicates identification, particularly when specimens exhibit intermediate or atypical hair coloration as explained in *X. ecuadorica* entry. Phylogenetic analyses based on multiple genetic markers support *X. piurensis* and *X. ecuadorica* as two different species (Melo & Martins, 2025). *X. incarum* was not included in the analysis. Future integrative taxonomic studies may help clarify species boundaries and identify more diagnostic traits to distinguish these taxa.

***Xylocopa* (*Schonnherria*) *viridigastra* Lepeletier, 1841**

*Xylocopa viridigastra* is distributed across the Neotropical region, including Chile, Ecuador, and Peru (Moure & Melo, 2023), occurring between sea level and 4,000 m a.s.l. (Ospina, 2000). In Ecuador, it is found throughout the Andes and inter-Andean valleys, from Cañar to Loja, as well as on both the eastern and western slopes, between 143 and 3,849 m a.s.l. (Fig. 4B). There is also an unusual record from Guayaquil at 3 m a.s.l., labeled as the nearest major city. Females of *X. viridigastra* are predominantly melanic with a faint green sheen. Males of *X. viridigastra* are characterized by enlarged compound eyes that are slightly convergent dorsally, a yellowish clypeus and paraocular areas, and predominantly black pubescence on the metasoma and legs. Whitish to yellowish hairs are restricted to the frontal area of the mesoscutum, the lateral regions around the tegulae, and the mesepisternum between the first and second pair of legs.

Confusion between *X. viridigastra* and *Neoxylocopa* females likely arose because both species are melanic. Identification keys describe *Schonnherria* species as having metasomal sterna that are smooth or only weakly marked by a longitudinal carina. In *X. viridigastra*, this ridge is more evident than in other members of the subgenus but remains less developed than in *Neoxylocopa*. Females of both species can be distinguished by the clypeus, which in *X. viridigastra* lacks a raised smooth ridge along the epistomal suture.

**DNA barcode.** The COI sequence (656 bp) obtained from the Ecuadorian specimen of *X. viridigastra* was assigned to BIN AGL1615. A second BIN, AAN9142, has also been reported for Ecuadorian *X. viridigastra* based on a COI-5P sequence of 579 bp, and this BIN has additionally been recorded from northern Chile (Table S4).

***Xylocopa* (*Schonnherria*) *viridis* Smith, 1854**

*Xylocopa viridis* is reported from Argentina, Bolivia, Brazil, Colombia, Costa Rica, French Guiana, Guatemala, Guyana, Mexico, Panama, Paraguay, and Peru (Moure & Melo, 2023), and is also listed for Ecuador (Ascher & Pickering, 2020). In Ecuador, it occurs in the northern and central Amazonian region between 217 and 986 m a.s.l. (Fig. 4C). *Xylocopa viridis* shows notable intraspecific variation across its range, including differences in body pubescence, punctation, body size, and the shape of the apical projection on the inner surface of the male hind tibia (Lucia, Gonzalez & Abrahamovich, 2015). From the specimens analyzed, both males and females of this species are characterized by green iridescence, pale yellowish-brown pubescence on the mesosoma, and mostly white to yellowish hairs on the hind basitarsi and metasomal terga, consistent with the keys of Villamizar, Fernández & Vivallo (2020), Mawdsley (2018) and Gonzalez, Gonzalez & Cuellar (2009). In the phylogenetic study of large Neotropical carpenter bees, only a single sequence of *X. viridis* was analyzed because the authors also considered that multiple species may be included under this name (Melo & Martins, 2025). These observations suggest that *X. viridis* likely represents a species complex, and further morphological and molecular studies are required to clarify species boundaries and determine whether one or more members of this group occur in Ecuador.

**DNA barcode.** The COI sequence (678 bp) was assigned to BIN AET4851 (Table S4), which is shared with *X. ecuadorica*. Despite clear morphological differences between the species, they cluster within the same BIN.

***Xylocopa* (*Schonnherria*)** **sp. 3**

All specimens examined were from Imbabura and the Carchi provinces at elevations between 1,655 and 2,562 m a.s.l. (Fig. 4D). Both sexes are characterized by a small body size and a black integument with bluish highlights on the head and mesosoma and greenish reflections on the metasoma. The wings are hyaline. Pubescence is predominantly black, with the legs entirely covered by similarly colored hairs. Females are distinguished by the labrum having a longitudinally elongated median tubercle at its base and by the terga showing short, simple black hairs on the discs that are barely noticeable. Males have enlarged compound eyes that are slightly convergent dorsally. The paraocular area and clypeus show yellowish pale coloration with long black hairs present.

***Xylocopa* (*Schonnherria*)** **sp. 4**

**Figure 5:**
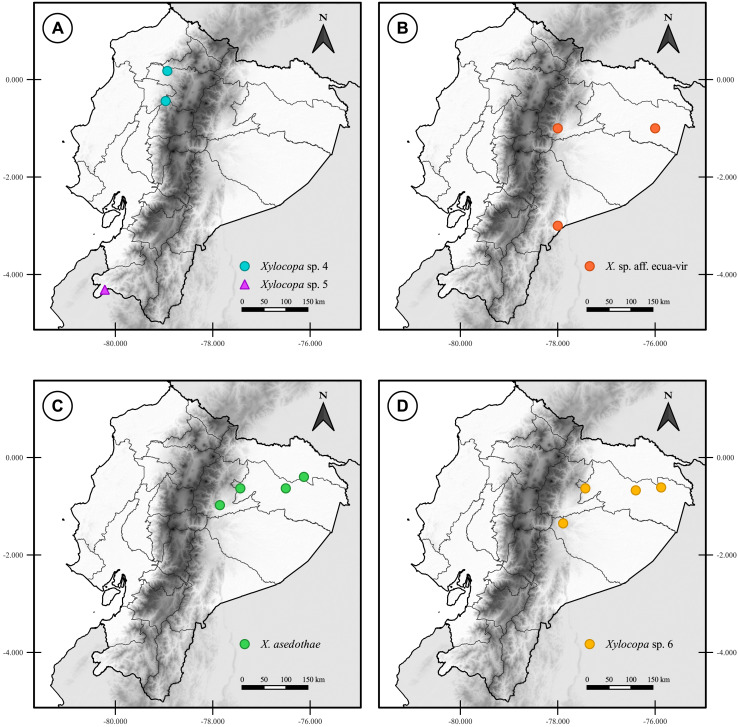
Distribution records. (A) *Xylocopa* (*Schonnherria*) sp. 4 and *Xylocopa* (*Schonnherria*) sp. 5; (B) *Xylocopa* (*Schonnherria*) sp. aff. *ecuadorica/viridis;* (C) *Xylocopa* (*Xylocopina*) *asedothae*; (D) *Xylocopa* (*Xylocopina*) sp. 6.

This species is represented in collections by a single large female from Cotopaxi Province (Otonga Reserve) at 1,591 m a.s.l. It has also been recorded from Mashpi Reserve, Pichincha Province, at 609 m a.s.l., based on a recent citizen science observation (Fig. 5A). The corresponding record is available on iNaturalist: 337398166. It is characterized by a metallic blue integument on the head and mesosoma and metallic green coloration on the metasoma, with reddish-brown areas on the posterior portions of the terga and sterna. Pubescence is mostly black, with white hairs present on the clypeus, paraocular areas, gena, the discs of T4–T5, and the lateral margins of T2–T5. The wings are dark brown with blue and violet iridescence. The base of the labrum bears a distinct, large, single tubercle. This species closely resembles *Xylocopa* (*Schonnherria*) *auriventris* Villamizar, Fernández & Vivallo, 2020 in overall morphology and diagnostic characters. However, the Ecuadorian specimen differs in having white hairs on the lateral margins of T2, whereas the original description of *X. auriventris* reports such hairs beginning on T3. *X. auriventris* was described from a single specimen from the Colombian Chocó, and photographs of the type show the pubescence appearing clumped, likely as a result of humidity.

***Xylocopa* (*Schonnherria*)** **sp. 5**

This species is represented by a single male in poor condition, housed at UNL and collected in Loja Province at 223 m a.s.l. (Fig. 5A). The specimen exhibits a metallic bluish integument and slightly convergent compound eyes, but lacks any pale or white coloration on the clypeus in contrast to all other males of *Schonnherria* examined in this study. Brown coloration is evident on the posterior sternites. Pubescence is predominantly dark, with longer hairs becoming apparent from T4 onward, and the legs are mostly covered with dark hairs.

**Subgenus *Xylocopina* *Hurd & Moure, 1963***

***Xylocopa* (*Xylocopina*) *asedothae* *Zama & Silveira, 2024***

*Xylocopa asedothae* was recently described from Colombia and Ecuador based solely on male specimens, representing the second known species of the subgenus *Xylocopina* (Zama & Silveira, 2024). Although *Xylocopina* was historically considered part of *Stenoxylocopa* Hurd & Moure, 1960, molecular analyses by Melo & Martins (2025) support its recognition as a distinct subgenus; however, only *X*. (*Xylocopina*) *ruficollis* Hurd & Moure, 1963 was included in that study. Future molecular data from *X. asedothae* will be essential to clarify the limits and monophyly of *Xylocopina*. New material analyzed here extends its known range eastward and confirms its presence across the northern Amazonian lowlands of Ecuador between 219 and 748 m a.s.l. (Fig. 5C). Males of *X. asedothae* can be recognized by their mostly yellow body hairs, densely hairy lower paraocular area, and almost clear wings (Zama & Silveira, 2024; Michener, 2007). To distinguish them from *Neoxylocopa* males, the integument is mostly black/dark brown except for the sides of the clypeus and paraocular area, forming a distinct dark inverted triangle on the clypeus and frons.

***Xylocopa* (*Xylocopina*)** **sp. 6**

*Xylocopa* (*Xylocopina*) sp. 6 is represented by five females collected in the Amazonian region of Ecuador, specifically in Orellana and Pastaza provinces, at elevations between 206 and 976 m a.s.l. (Fig. 5D). These specimens are entirely melanic, with a black integument and completely black tegulae. The mandibles are tridentate, and the clypeus has distinctly raised margins. Pubescence is predominantly black on both the mesosoma and metasoma. The wings are dark brown with a faint violet iridescence. The sterna lack a mid-longitudinal carina, and the hind tibia has a pair of spines at the outer apical margin. Given their occurrence in the same region this morphospecies could be the unknown female of *X. asedothae*. However, due to the sexual dimorphism, this hypothesis requires confirmation through molecular analyses and additional ecological or behavioral evidence.

**DNA barcode.** The COI sequence (678 bp) was assigned to BIN AHI1192 (Table S4).

### Carpenter bees and their interacting plants

**Figure 6:**
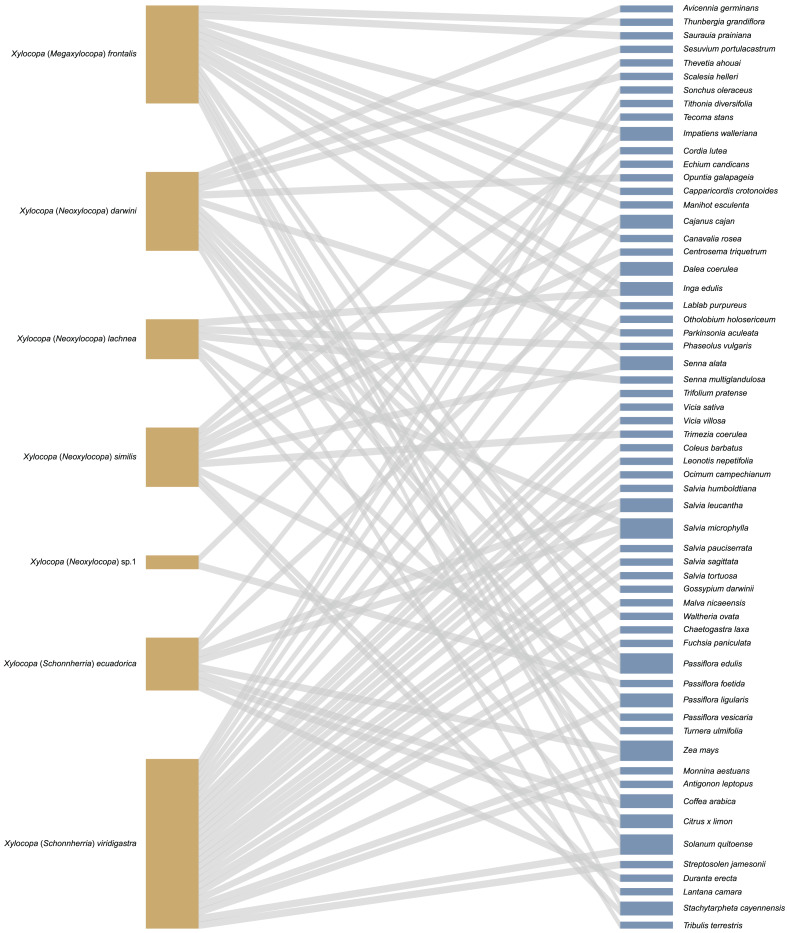
Binary species–species interaction network of Ecuadorian carpenter bees. Binary interaction network showing Ecuadorian carpenter bees (*Xylocopa*) and their associated flowering plant species. Links indicate the presence of an interaction. The network was constructed using the bipartite package (Dormann, Gruber & Fründ, 2008) in R (v. 4.3.1).

Our dataset includes 188 recorded interactions (Table S2). Of these, 172 were identified to species level (Fig. 6), involving seven *Xylocopa* species, 26 plant families, and 60 plant species. Notably, 34 of the plant species were documented in only a single interaction. Overall, 28 plant species are native, six are endemic, and 26 are exotic. It is important to note that identifications based solely on photographs may be uncertain, as some plant species are difficult to distinguish without physical examination. We documented 110 plant–bee interactions from iNaturalist records (Table S2), involving six species of carpenter bees (*Xylocopa*), associated with 23 plant families and 44 plant species, 28 of which were singletons. Among these plant species, 17 are native, five are endemic, and 22 are exotic. From museum labels, we recorded 78 additional interactions (Table S2), representing seven *Xylocopa* species, 11 plant families, and 20 plant species, including 10 singletons; 13 species are native, one is endemic, and six are exotic.

**Figure 7:**
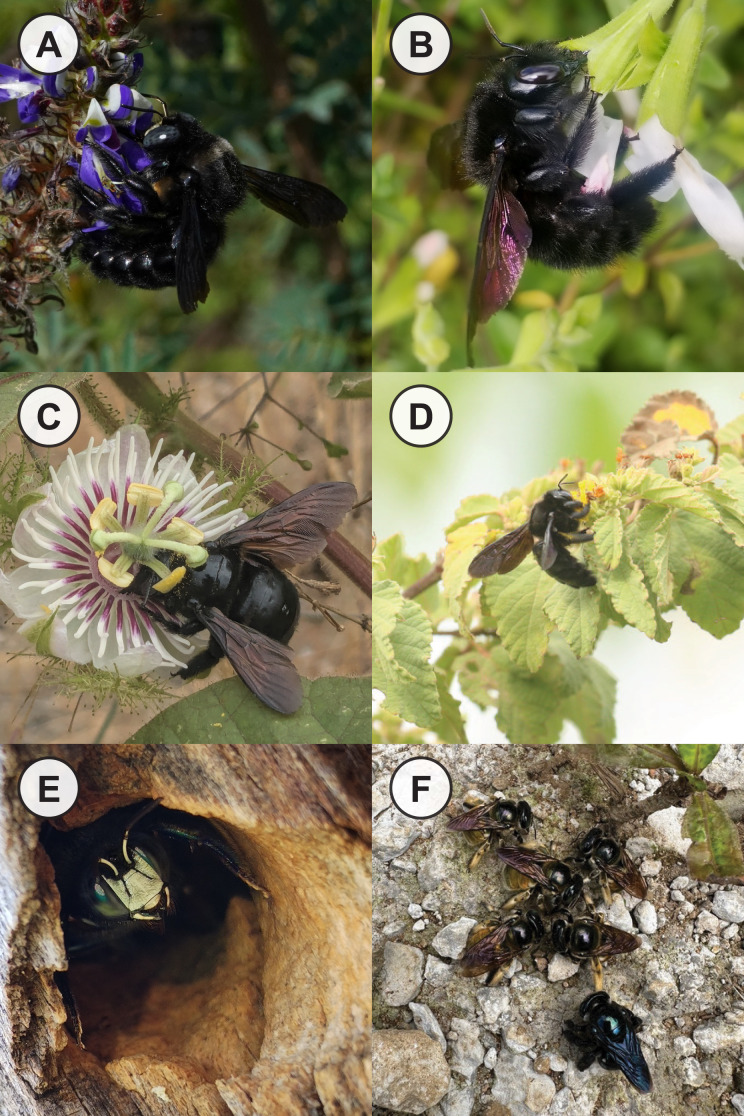
Examples of species carpenter bees (*Xylocopa*) present in Ecuador documented *via* iNaturalist. (A) *Xylocopa viridigastra* (male) on *Dalea coerulea* (credit: Esteban Poveda, user: quilicotriste, iNaturalist: 341398205). (B) *Xylocopa lachnea* (female) on *Salvia microphylla* (credit: Esteban Poveda, user: quilicotriste, iNaturalist: 294080690). (C) *Xylocopa* (*Neoxylocopa*) sp. 1 (female) on *Passiflora foetida* (credit: Raul Ontaneda, user: raul9910, iNaturalist: 307756720). (D) *Xylocopa darwini* (female) on *Waltheria ovata* (credit: Philip Precey, user: philip_precey, iNaturalist: 250838871). (E) *Xylocopa ecuadorica* (male) nesting on *Schinus molle* (credit: Esteban Poveda, user: quilicotriste, iNaturalist: 321892858). (F) *Xylocopa ornata* (female, middle) and *Xylocopa lucida* (female, down) collecting salts from the ground (credit: Ferhat Gundogdu, user: heimatlos, iNaturalist: 309376356). Photographs not taken by the authors are released under a CC0 license on iNaturalist, and the photographers were contacted and informed of their use in this publication.

The species with the highest number of recorded interactions was *X. viridigastra* (Fig. 7A), associated with 26 plant species and representing 43% of all records. Of these, 11 species are native and 2 are endemic. This pattern aligns with museum data, where *X. viridigastra* accounts for 36% of all examined specimens; however, the higher number of interactions likely reflects its larger number of records and potential collection bias rather than true relative abundance. The second most frequently recorded species was *X. frontalis*, associated with 15 plant species, 8 of which are native. Next was *X. darwini* (Fig. 7D), which interacted with 12 plant species, 4 of them are endemic, reflecting the species’ occurrence in the Galápagos, where endemism rates are higher. *X. similis* followed with nine recorded interactions, five involving native plant species; *X. ecuadorica* (Fig. 7E) with eight species, two native; and *X. lachnea* (Fig. 7B) with six species, four native. Finally, *Xylocopa* sp. 1 (Fig. 7C) was represented only by two museum records involving native plants, both documented by the authors.

The plant family visited by the greatest number of *Xylocopa* species (Fig. S1) was Fabaceae (Fig. 7A), recorded for six of the seven species, followed by Passifloraceae (Fig. 7C), visited by five carpenter bee species. Families Lamiaceae (Fig. 7B), Poaceae, Solanaceae, Verbenaceae, and Asteraceae were each visited by three species. The plant species associated with the highest number of *Xylocopa* species, as indicated by the interaction network (Fig. 6), were the native *Solanum quitoense* (naranjilla) and the exotics *Salvia microphylla* (baby sage; salvia) (Fig. 7B), *Zea mays* (maize; choclo) and *Passiflora edulis* (passion fruit; maracuyá).

The species–plant origin network, showing the intensity of interactions per plant origin, is provided as Fig. S2.

### DNA barcoding and tree construction

**Figure 8:**
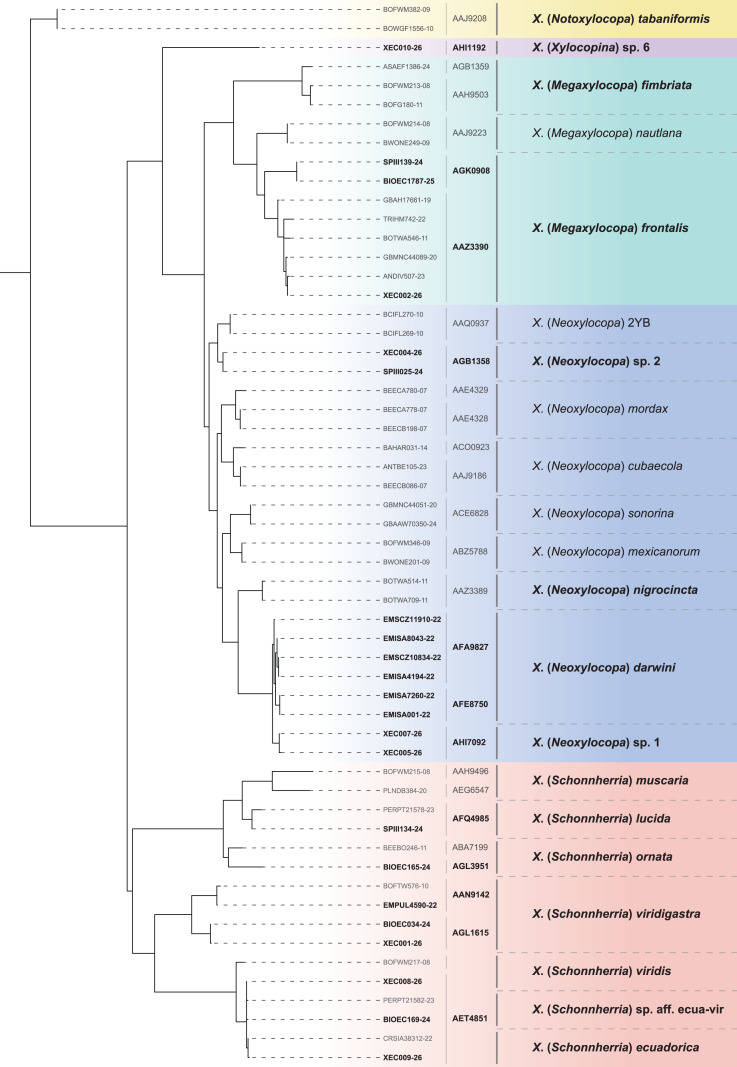
Maximum Likelihood phylogeny of *Xylocopa* based on COI sequences, including species recorded from Ecuador and additional reference taxa. Sequences collected in Ecuador are indicated in bold, as are species recorded for the country. Colors denote subgenera (from top to bottom): yellow, *Notoxylocopa*; purple, *Xylocopina*; cyan, *Megaxylocopa*; blue, *Neoxylocopa*; and red, *Schonnherria*. Node support values and outgroup information are provided in Figs. S3 and S4.

The maximum likelihood (ML) phylogeny recovered all five subgenera as monophyletic (Fig. 8), including *Megaxylocopa* (aLRT = 92.5; UFBoot = 90), *Neoxylocopa* (aLRT = 83.8; UFBoot = 71), *Schonnherria* (aLRT = 75.8; UFBoot = 81), *Xylocopina* (aLRT = 92.2; UFBoot = 65), and *Notoxylocopa* (aLRT = 65; UFBoot = 89). *Xylocopa* (*Proxylocopa*) *olivieri* Lepeletier, 1841 was correctly placed as the outgroup. The topology was congruent with the phylogenetic framework proposed by Melo & Martins (2025) and supports the utility of the COI barcode region for resolving subgeneric relationships within Neotropical *Xylocopa*. Complete bootstrap and SH-like aLRT support values for all nodes are shown in Figs. S3 and S4.

***Megaxylocopa*.-** *Xylocopa frontalis* was recovered as monophyletic and exhibited relatively high intraspecific K2P divergence (mean = 3.40%, range = 0.00–6.06%). The phylogeny revealed two well-differentiated lineages associated with geographic regions: an Amazonian clade (aLRT = 78.3; UFBoot = 58) showing moderate internal divergence (mean = 2.02%, range = 0.47–2.89%), and a Pacific coastal clade (aLRT = 98.6; UFBoot = 100). The genetic distance between these clades (mean = 5.41%, range = 4.47–6.06%) exceeded the interspecific divergence between *X. frontalis* and its closest relative, *X. nautlana* (mean = 4.69%, range = 4.05–5.53%), indicating strong geographic structuring between these two populations.

These results, together with the morphological differences we observed between South American and Mesoamerican males of *X. frontalis*, suggest that taxonomic boundaries within this species may require reassessment. A comprehensive revision across the species’ full distributional range, integrating morphological and molecular data, is warranted.

***Neoxylocopa*.-** *Xylocopa darwini* and *Xylocopa* sp. 1 formed a strongly supported clade (aLRT = 98.4; UFBoot = 100). Their interspecific K2P distance (mean = 2.14%, range = 1.57–2.66%) was markedly lower than the intraspecific inter-island divergences observed in the Caribbean island species *Xylocopa mordax* Smith, 1874 (mean = 6.31%, range = 5.87–6.73%) and *Xylocopa cubaecola* Lucas, 1857 (mean = 6.79%, range = 6.21–8.11%). This close genetic relationship, together with the low level of morphological differentiation between *X. darwini* and *Xylocopa* sp. 1, suggests that they likely represent sister lineages, with one occurring on the mainland and the other in the Galápagos Archipelago. Further integrative taxonomic and biogeographic studies are needed to clarify species boundaries and to refine estimates of the timing and patterns of colonization of the archipelago from the continent. Phylogeographic patterns in *X. darwini* show historical isolation among islands, with an initial colonization of central and western islands and subsequent eastward range expansion under limited gene flow (Vargas et al., 2015).

*Xylocopa* sp. 2 was recovered as sister to the unidentified *X*. (*Neoxylocopa*) 2YB from Panama, forming a well-supported clade (aLRT = 85; UFBoot = 71) with an interspecific distance of 2.99%. The occurrence of both *X*. 2YB and *X*. *columbiensis* in Panama, together with the morphological similarity between *X*. sp. 2 and *X*. *columbiensis*, raises the possibility that *X*. 2YB corresponds to *X*. *columbiensis*, with *Xylocopa* sp. 2 representing a closely related, potentially undescribed taxon. However, without access to type material or high-quality comparative images, a formal identification could not be confirmed. The marked molecular differentiation from *Xylocopa mexicanorum* Cockerell, 1912 (mean = 4.75–4.86%) and *Xylocopa sonorina* (mean = 4.23–4.86%), suggests the presence of an distinct genetic lineage *Neoxylocopa* species group not represented in current identification keys for Mesoamerica. Clarifying the taxonomic status of these lineages will require coordinated integrative studies combining morphological and molecular data from across the region.

***Schonnherria*.-** Molecular analyses consistently grouped *X. ecuadorica* and *X. viridis* together, forming a strongly supported clade (aLRT = 99.3, UFBoot = 100). *Xylocopa ecuadorica*, which occurs along the Pacific coast and Andes, showed low intraspecific divergence (0.46%), while the interspecific distance between the Ecuadorian *X. viridis* specimen (XEC008-26) and *X. ecuadorica* was even lower (0.37%). This indicates that the COI barcode alone is insufficient to reliably separate these taxa and highlights the need for an integrative taxonomic assessment. Specimens with intermediate morphology traits between *X. ecuadorica* and *X. viridis*, tentatively referred to as *Xylocopa sp. aff. ecuadorica/viridis* (abbreviated in figures as *Xylocopa sp. aff. ecua–vir*), are described in detail in Article S1. These specimens, collected from the Ecuadorian and Peruvian Amazon, where they co-occur with *X. viridis*, show K2P distances close to both *X. ecuadorica* (mean = 0.54%, range = 0.31–0.77%) and *X. viridis* (mean = 0.15%), reflecting the complexity within this clade.

This pattern may reflect recent divergence with incomplete lineage sorting, historical introgression, past hybridization, or alternatively a single, widely distributed species with considarable intraspecific variation. In contrast, the Mexican *X. viridis* specimen (BOFWM217-08) was outside this clade and differed from Ecuadorian *X. viridis* (3.54%), consistent with the idea that *X. viridis* likely represents a species complex rather than a single species. These results highlight the need for additional nuclear markers and broader sampling across the full geographic and morphological range of these taxa.

*Xylocopa muscaria* showed high divergence, with the two sampled specimens from Costa Rica and Mexico exhibiting a K2P distance of 10.70%, exceeding all interspecific comparisons in this dataset. Photographs associated with these specimens in BOLD are inconsistent with the description of *X. muscaria* in our study, particularly regarding complete genal setation, raising doubts about their correct identification. Together, these results suggest that the current concept of *X. muscaria* may encompass multiple distinct genetic lineages that could correspond to more than one species, indicating the need for a thorough taxonomic revision.

*Xylocopa viridigastra* also showed intraspecific divergence (mean = 3.69%, range = 0.46–5.72%), with the Ecuadorian specimen from Pichincha (EMPUL4590-22) being substantially closer to a Chilean specimen (1.04%) than to other Ecuadorian specimens (5.03–5.72%), suggesting possible geographic structure or cryptic diversity within this species across its Andean distribution. *Xylocopa ornata* exhibited a divergence of 4.71% between Ecuadorian and Bolivian specimens, which warrants further investigation given the broad Amazonian range of this taxon. In contrast, *Xylocopa lucida* showed a more moderate divergence of 1.24% between Ecuadorian and Peruvian specimens, consistent with a single, continuously distributed species on both sides of the Andes.

## Discussion

This study represents the first comprehensive assessment of *Xylocopa* in continental Ecuador. We confirmed the presence of *X. ornata*, *X. metallica*, and *X. nigrocincta*, previously unreported in the country (Moure & Melo, 2023; Ascher & Pickering, 2020; Villamizar, Fernández & Vivallo, 2020; Ospina, 2000; Hurd, 1978). Despite this progress, taxonomic challenges remain a significant limitation in understanding *Xylocopa* diversity (Agostini et al., 2024; Mawdsley, 2017). Specimens from five morphotypes could not be confidently assigned to any described species, even after applying all available identification keys, species descriptions, and reference photographs. These specimens likely represent taxa not yet formally documented or described, the absence of confirmed new species in this catalogue should not be interpreted as evidence of absence. Continued efforts in taxonomy, specimen curation, and the development of modern identification and digitization tools are essential to improve the quality, accessibility, and completeness of biodiversity data (Marcer et al., 2022; Kharouba et al., 2019), particularly in regions like Ecuador where under-sampling and gaps in publicly available occurrence records likely lead to underestimates of true species richness (Boyd et al., 2022; Freitas et al., 2009). Additional studies combining fieldwork, taxonomic and molecular reviews are needed to better document the diversity and distribution of bees in the region.

Intraspecific variation in coloration was evident in several *Xylocopa* species in Ecuador. In the Amazon, *X. frontalis* and *X. nigrocincta* display a red-banded morphotype, whereas completely dark morphs occur in other regions. *X. ecuadorica* also exhibited color variation, with some individuals showing more reddish hairs. Such polymorphisms are common in tropical insects and are often associated with ecological and selective pressures (Agostini et al., 2024; Lucia & Gonzalez, 2017; Hill et al., 2012). Although some *Xylocopa* species show high dispersal capacity and gene flow (Cont et al., 2024; González-Cordoba & Montoya-Lerma, 2014), regional differences in color morphs may still be maintained by local selective pressures. Environmental conditions, particularly climate and light availability, may influence coloration through their effects on thermal regulation and light-related selection pressures (Haque, Khan & Herberstein, 2024; Kozlov et al., 2022; Suni & Dela Cruz, 2021). Predation may also contribute to these patterns, as coloration that improves camouflage or reduces detectability can increase survival (Zvereva et al., 2019). In addition, sexual selection may favor particular morphs involved in mate recognition or courtship displays, promoting divergence in coloration (Aslam, Nedvěd & Sloggett, 2024; Blaimer, Mawdsley & Brady, 2018; Leys & Hogendoorn, 2008). Developmental factors may further influence this variation, since diet during the larval stage can affect pigmentation through differences in floral resources or nutrient intake (Liao et al., 2025; Davis, 2014). Because multiple mechanisms may shape these patterns, documenting the geographic distribution of color morphs is important for understanding their origin and maintenance. Citizen science initiatives provide a useful approach for tracking such variation across regions and complement traditional field surveys (Lehtinen et al., 2020) as seen in this study with *X. frontalis*. The presence of multiple color morphs within a single species can also complicate species delimitation, since morphological differences that appear distinct at first glance may represent intraspecific variation rather than separate species, potentially leading to misidentification or overestimation of bee diversity (Agostini et al., 2024; Freitas et al., 2018; Ferrari & Melo, 2014).

Dispersal capability influences the distribution and connectivity of *Xylocopa* in Ecuador. Some species, including *X. frontalis*, *X. lachnea*, and *X. ecuadorica*, may occasionally cross low-elevation sections such as the Huancabamba Depression. As in this area, these species occur in the inter-Andean valleys and on both the eastern and western slopes. The cordillera highest point (~2,100 m) falls within their altitudinal range. Similar cross-Andean movements have been documented in several taxa within the Amotape–Huancabamba Zone, where low passes enable trans-Andean dispersal (Weigend, 2002; Duellman et al., 1979). Further studies are needed to evaluate the extent to which this corridor influences gene flow and population structure in *Xylocopa*. While this may reflect possible natural dispersal, human-mediated movement also shapes *Xylocopa* distributions. Transport of wood products can bypass natural barriers, spreading wood-nesting bees and potentially introducing non-native species or altering native populations (Fenn-Moltu et al., 2023; Poulsen & Rasmussen, 2020; Ruiz et al., 2020). For example, *Xylocopa* sp. 1 has been found in the United States *via* imported Ecuadorian balsa wood (*Ochroma pyramidale*) (USNMENT01613529). Similarly, *X. sonorina* has been introduced to several Pacific islands (Sheffield, Heron & Musetti, 2020; Hurd, 1958), illustrating how human-assisted dispersal can facilitate the spread of wood-nesting bees beyond their native ranges. Such movement poses a threat to native species in coastal Ecuador, as well as to the endemic *X. darwini* in the Galápagos. Preventing the spread of exotic wood-nesting insects is challenging due to the extensive and largely unregulated lumber trade within the country. Overall, dispersal patterns of bees in Ecuador remain largely unstudied, representing a critical gap for conservation research.

Recent global declines in insect populations, driven by habitat loss, pesticide use, climate change, and other anthropogenic pressures (Hailay Gebremariam, 2024; Wagner, 2019), underscore the urgency of conserving pollinators. Despite this, no *Xylocopa* species in the Americas have been assessed by the IUCN (2025). Major knowledge gaps remain regarding their ecology, population dynamics, and conservation status (Rousseau et al., 2024). Temperature change may further threaten *Xylocopa* populations, as extreme temperatures can reduce their survival and reduce pollination activity (De Farias-Silva & Freitas, 2021; Kachhawa, Charan & Nagar, 2020). Protecting these bees is therefore critical not only for sustaining ecosystem functioning and crop productivity but also for safeguarding genetic and species diversity (Valverde et al., 2024; Ramos-Jiliberto, Moisset De Espanés & Vázquez, 2020).

The documented interactions show that *Xylocopa* visits a wide range of floral resources, including both native and exotic species. While some visits likely result in pollination, others involve nectar robbery (Fig. 7B), producing variable reproductive outcomes for plants (Shaw-Bonner et al., 2023; Wang et al., 2023). Consequently, the interactions documented in this study should not be interpreted as evidence of effective pollination. Overall, the species of carpenter bees from Ecuador behave as generalist floral visitors, exploiting diverse flowers rather than specializing (Ferreira et al., 2024a; Chamorro et al., 2012). Notably, they also visited endangered endemic species such as *Otholobium holosericeum* (Neill & Pitman, 2004), highlighting their potential role in supporting vulnerable plants. This interaction was documented at the Cuenca Botanical Garden, involving a male *X. viridigastra*. The results of the documented interactions showed that the three most frequently visited species are crops or ornamentals, indicating that *Xylocopa* plays a role in human-modified landscapes (Junqueira & Augusto, 2017; Vinícius-Silva et al., 2017). Nevertheless, this higher number of records may be explained by the abundance, accessibility, and frequent observation of these plants through citizen science or their study for economic purposes. Interactions with *Zea mays* (Poaceae), a wind-pollinated species, suggest that *Xylocopa* may exploit wind-pollinated plants as a low nutrient pollen source. This behavior is well-documented in bees visiting anemophilous species across a wide range of habitats (Abrahamczyk, Struck & Weigend, 2023; Danner, Härtel & Steffan-Dewenter, 2014; Höcherl et al., 2012). At the same time, interactions with exotic species suggest that *Xylocopa* may aid the spread of non-native plants, demonstrating both positive and negative ecological consequences of their generalist foraging. Species-level patterns should not be concluded from this study because, for example, *X. viridigastra* is particularly common near Quito, leading to higher sampling and photographic frequency on citizen science platforms, while identifying any of the melanic *Neoxylocopa* species from such records is challenging due to the strong morphological similarity among females.

Because the interaction dataset compiled here integrates museum records, literature reports, online biodiversity databases, and citizen science observations such as iNaturalist, it reflects heterogeneous sampling effort rather than a standardized ecological survey (Soteropoulos, De Bellis & Witsell, 2021). Sampling of tropical bees remains uneven, particularly in regions of the Global South where field studies and taxonomic knowledge are still limited (Orr et al., 2021; Freitas et al., 2009). Bee–plant interaction data are especially scarce for most Neotropical species, restricting the ability to infer ecological specialization or pollination effectiveness. Additionally, citizen science observations tend to be concentrated in accessible locations and may overrepresent conspicuous species and frequently cultivated plants (Geurts, Reynolds & Starzomski, 2023). Consequently, the interactions documented here should be interpreted as a preliminary synthesis of currently available records rather than a complete representation of *Xylocopa* floral associations in Ecuador, highlighting the need for targeted ecological surveys and long-term monitoring.

## Conclusions

Our study represents the first assessment of carpenter bees (*Xylocopa*) in Ecuador, combining museum specimens, field surveys, citizen science observations, and DNA barcoding. We confirmed the presence of 16 species and six unassigned morphospecies, with seven of them visiting 60 plant species spanning native, endemic, and exotic taxa. These bees act primarily as generalist floral visitors, engaging in both legitimate pollination and nectar-robbing. Citizen science data were particularly valuable for expanding geographic and ecological coverage, complementing traditional collection records. Future efforts integrating molecular research, ecological studies, and community-based monitoring will be critical for clarifying species boundaries, understanding ecological roles, and informing strategies to conserve Ecuador’s diverse carpenter bee fauna.

## Supplemental Information

10.7717/peerj.21345/supp-1 Supplemental Information 1 Weighted species–family interaction network of Ecuadorian carpenter bees. Interaction network showing Ecuadorian carpenter bees (*Xylocopa*) and the plant families they visit. Links are weighted by the number of plant species per family that each bee species interacts with. The network was constructed using the bipartite package (Dormann et al., 2014) in R (v. 4.3.1).

10.7717/peerj.21345/supp-2 Supplemental Information 2 Weighted species–plant origin interaction network of Ecuadorian carpenter bees. Interaction network showing Ecuadorian carpenter bees (*Xylocopa*) and flowering plants grouped by origin (native, endemic, or exotic). Links are weighted by the number of plant species in each origin category that each bee species interacts with. The network was constructed using the bipartite package (Dormann et al., 2014) in R (v. 4.3.1).

10.7717/peerj.21345/supp-3 Supplemental Information 3 Maximum Likelihood tree of Ecuadorian *Xylocopa* based on COI sequences, including only sequences collected in Ecuador. Node support values are shown as ultrafast bootstrap percentages/SH-like aLRT values above each branch. Branch lengths are proportional to substitutions per site. The outgroup is *Xylocopa (Proxylocopa) olivieri* Lepeletier, 1841.

10.7717/peerj.21345/supp-4 Supplemental Information 4 Maximum Likelihood tree of *Xylocopa* based on COI sequences, including Ecuadorian sequences, sequences of species reported for Ecuador but collected abroad, and additional reference sequences. Node support values are shown as ultrafast bootstrap percentages/SH-like aLRT values above each branch. Branch lengths are proportional to substitutions per site. The outgroup is **Xylocopa* (Proxylocopa) olivieri* Lepeletier, 1841.

10.7717/peerj.21345/supp-5 Supplemental Information 5 Occurrence records, plant interactions, DNA barcode data, and genetic distances for *Xylocopa* species in Ecuador. This file contains Supplementary Tables S1–S6, including verified occurrence records, insect–plant interactions, specimen and DNA barcode data, Barcode Index Numbers (BINs), genetic distance matrices, and excluded records of *Xylocopa* species from Ecuador, with each dataset provided in a separate worksheet and described in its corresponding table legend.

10.7717/peerj.21345/supp-6 Supplemental Information 6 Ecology and taxonomic notes on some Ecuadorian carpenter bees (*Xylocopa*), with excluded species from the national checklist. This supplemental article presents ecological and behavioral observations of Ecuadorian *Xylocopa*, including nesting, non-floral resource use, interspecific interactions, and biogeographical context. It also provides species excluded from the checklist due to misidentification, taxonomic, or geographical uncertainty.

10.7717/peerj.21345/supp-7 Supplemental Information 7 COI 5P barcode sequences of *Xylocopa* species used in phylogenetic analyses. FASTA file containing 57 COI 5P barcode sequences of *Xylocopa* species, including specimens collected in Ecuador, specimens of species reported for Ecuador but collected outside the country, and additional reference sequences. Sequence names correspond to BOLD process IDs.

10.7717/peerj.21345/supp-8 Supplemental Information 8 R script for bipartite network construction and genetic distance estimation. R script (v 4.3.1) used to construct and visualize three bipartite interaction networks between *Xylocopa* species and plant resources (by plant species, plant family, and plant origin status) using the bipartite package, and to calculate pairwise Kimura 2-Parameter (K2P) genetic distances from the COI alignment using the ape package. Input files are Table S2 and Data S1.

## Additional Information and Declarations

### Field Study Permissions

The following information was supplied relating to field study approvals (*i.e*., approving body and any reference numbers):

The research was authorized by the Ministerio del Ambiente, Agua y Transición Ecológica (MAATE) of Ecuador. Permits were issued to Pontificia Universidad Católica del Ecuador (PUCE) and the Instituto Nacional de Biodiversidad (INABIO) (MAATE-DBI-CM-2024-0440 (PUCE) and MAATE-DBI-CM-2023-0309 (INABIO)).

### DNA Deposition

The following information was supplied regarding the deposition of DNA sequences:

The DNA barcode sequences generated in this study are available in the Barcode of Life Data System (BOLD): DS-XYLOEC, Carpenter Bees (Apidae: Xylocopa) of Ecuador; https://dx.doi.org/10.5883/DS-XYLOEC.

The sequences are also available in the Supplemental Files.

### Data Availability

The following information was supplied regarding data availability:

The raw data and code are available in the Supplemental Files.
