# Supplementary figures and images for: Carpenter bees (Apidae: Xylocopa) of Ecuador: distribution, DNA barcodes and plant interactions
Source: PeerJ. 2026 Jun 16;14:e21345. doi: 10.7717/peerj.21345 (PMC13281749; doi:10.7717/peerj.21345)

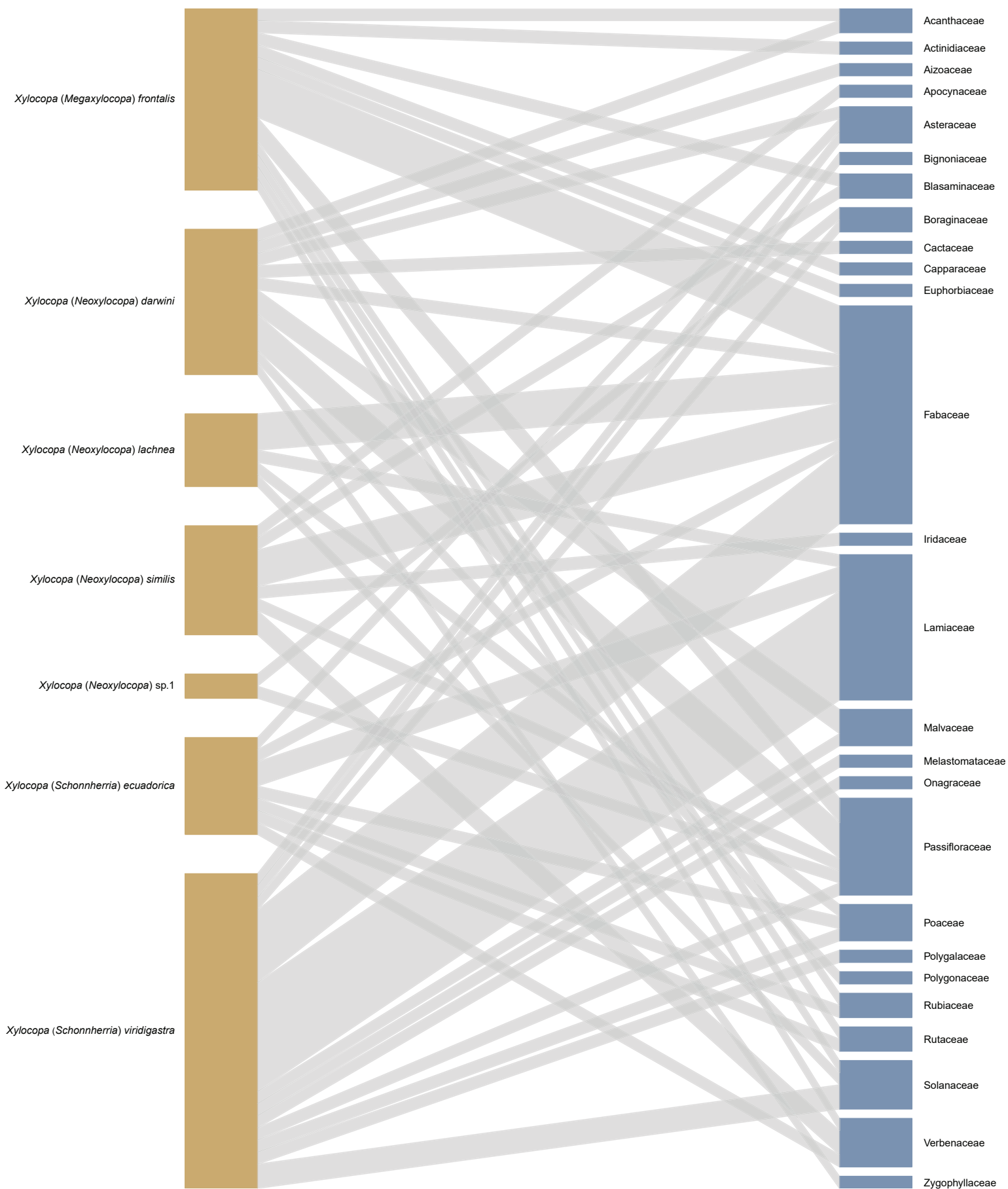

Supplement: Supplemental Information 1 — Interaction network showing Ecuadorian carpenter bees (Xylocopa) and the plant families they visit. Links are weighted by the number of plant species per family that each bee species interacts with. The network was constructed using the bipartite package (Dormann et al., 2014) in R (v. 4.3.1). [file peerj-14-21345-s001.pdf]

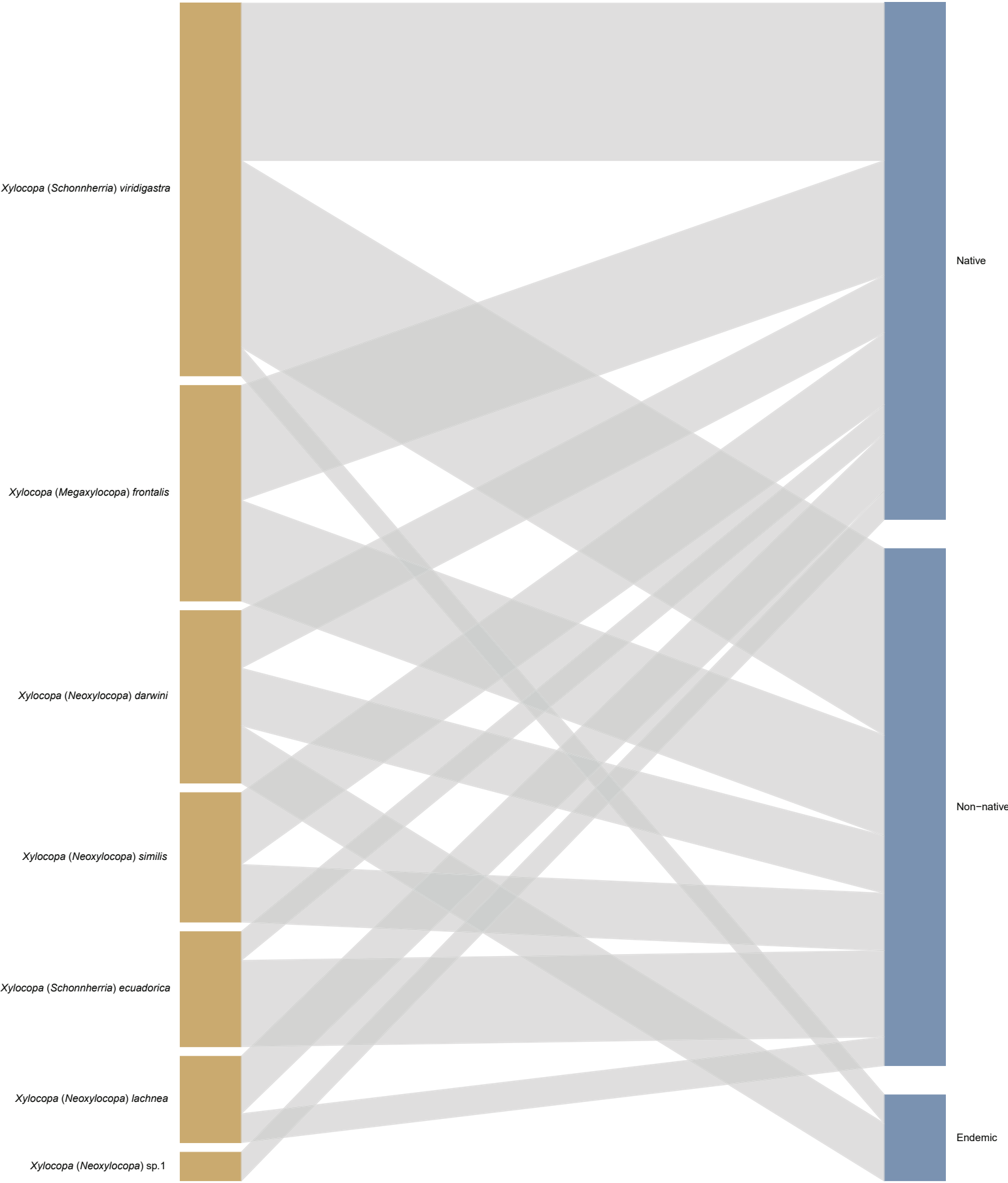

Supplement: Supplemental Information 2 — Interaction network showing Ecuadorian carpenter bees (Xylocopa) and flowering plants grouped by origin (native, endemic, or exotic). Links are weighted by the number of plant species in each origin category that each bee species interacts with. The network was constructed using the bipartite package (Dormann et al., 2014) in R (v. 4.3.1). [file peerj-14-21345-s002.pdf]

Tree scale: 1

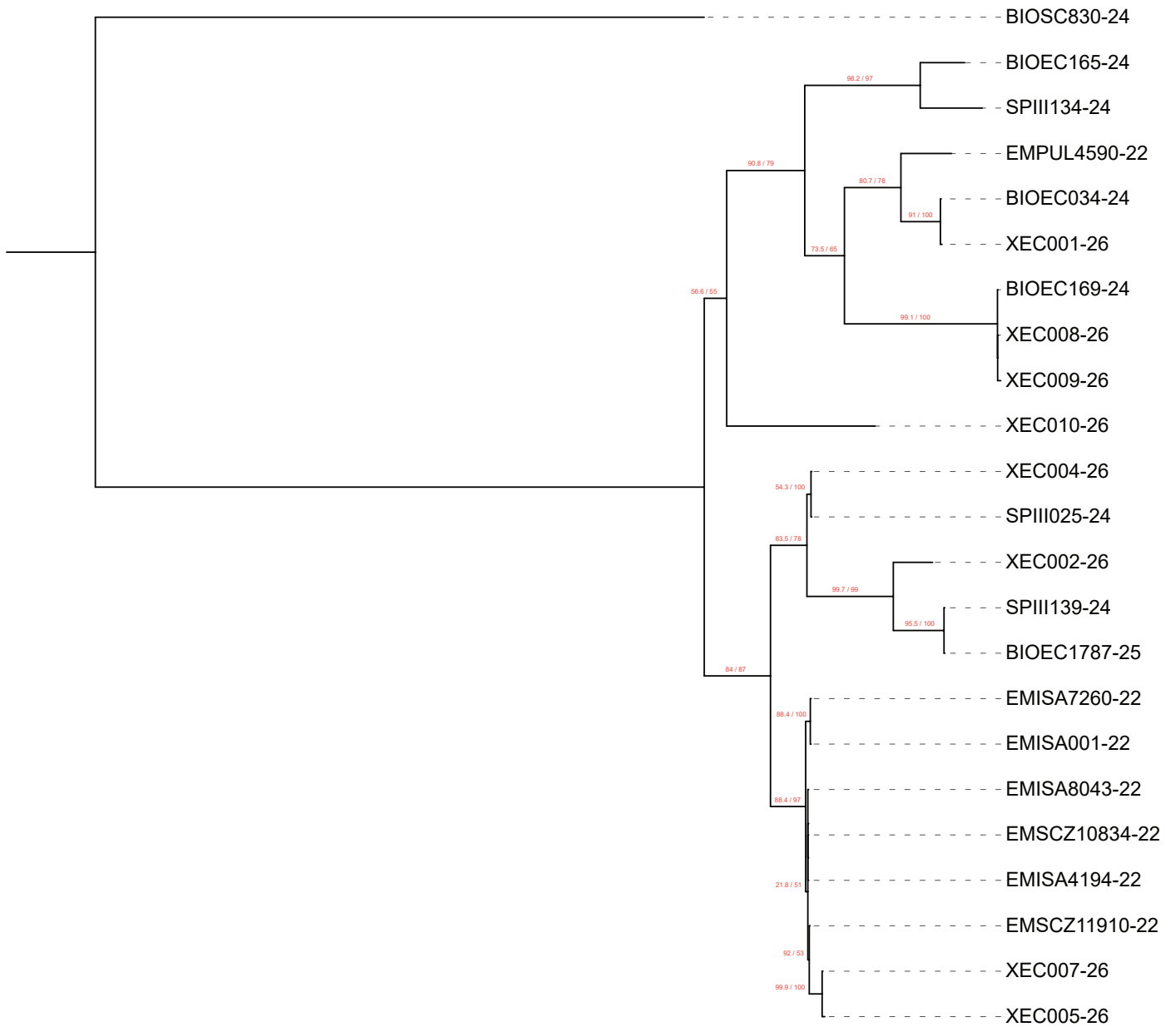

Supplement: Supplemental Information 3 — Node support values are shown as ultrafast bootstrap percentages/SH-like aLRT values above each branch. Branch lengths are proportional to substitutions per site. The outgroup is Xylocopa (Proxylocopa) olivieri Lepeletier, 1841. [file peerj-14-21345-s003.pdf]

Tree scale: 1

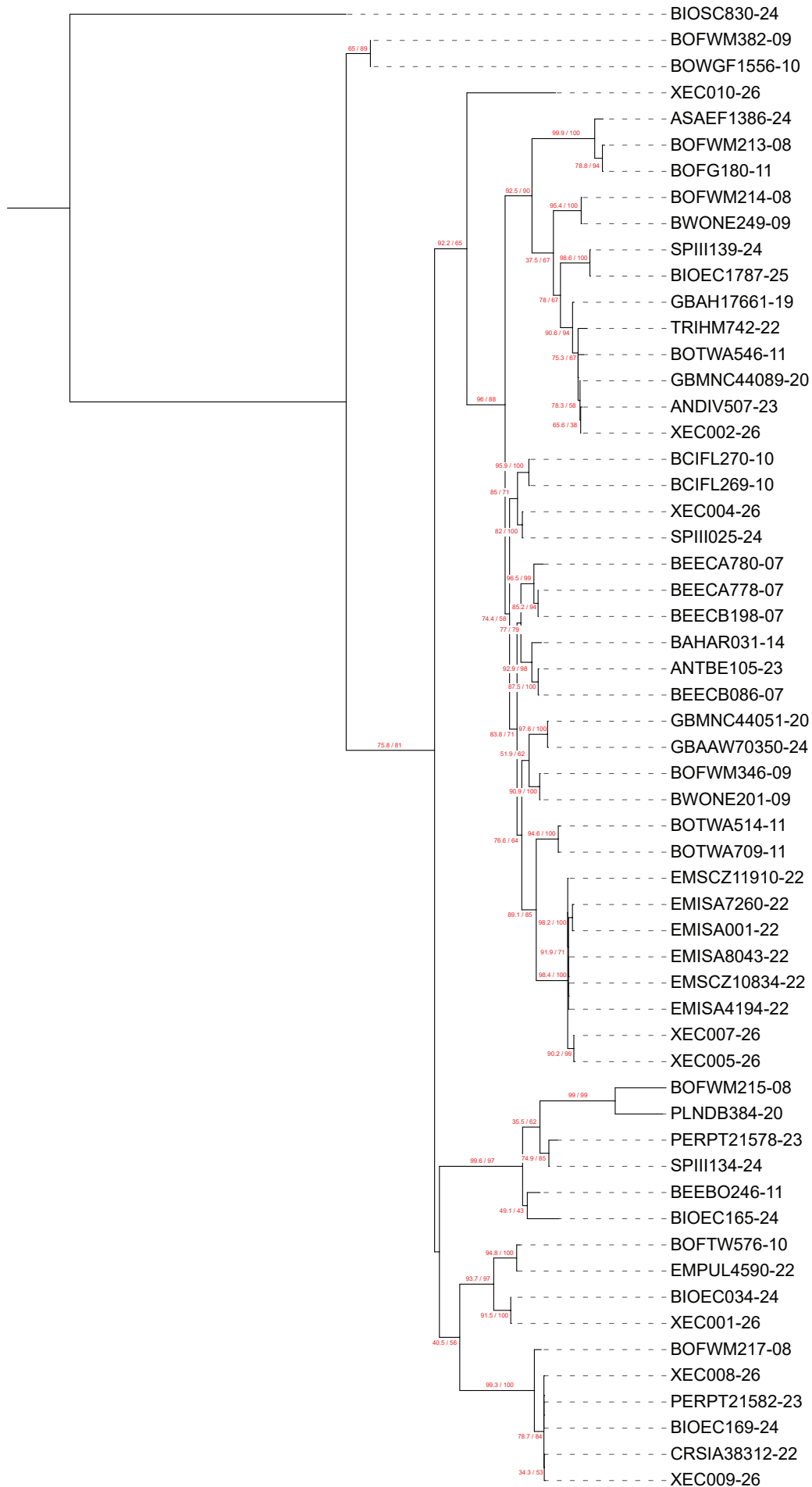

Supplement: Supplemental Information 4 — Node support values are shown as ultrafast bootstrap percentages/SH-like aLRT values above each branch. Branch lengths are proportional to substitutions per site. The outgroup is Xylocopa (Proxylocopa) olivieri Lepeletier, 1841. [file peerj-14-21345-s004.pdf]
