# Supplementary material for: Carpenter bees (Apidae: Xylocopa) of Ecuador: distribution, DNA barcodes and plant interactions
Source: PeerJ. 2026 Jun 16;14:e21345. doi: 10.7717/peerj.21345 (PMC13281749; doi:10.7717/peerj.21345)
Supplement: Supplemental Information 6 — This supplemental article presents ecological and behavioral observations of Ecuadorian Xylocopa, including nesting, non-floral resource use, interspecific interactions, and biogeographical context. It also provides species excluded from the checklist due to misidentification, taxonomic, or geographical uncertainty. [file peerj-14-21345-s006.docx]

**Supplemental Article 1**

**Carpenter Bees (Apidae: *Xylocopa*) of Ecuador: Distribution, DNA Barcodes and Plant Interactions**

Raul Ontaneda-Gallegos^1*†,^ Emilia A Moreno-Coellar^1,2,3*†^, Ana B. García-Ruilova^3^, Fernanda Salazar-Buenaño^1^, Esteban Poveda-Proaño^1^, Daniela Reyes-Barriga^3^, Pamela Lojan-Cueva^3^, Melanie Polo-Calva^3^, David A. Donoso^4^ & Álvaro Barragán^1^

^1^ Centro de Investigaciones de la Biodiversidad (CIBIO), Museo QCAZ Invertebrados, Pontificia Universidad Católica del Ecuador (PUCE), Quito, Ecuador

^2^ Grupo de Fisiología del Comportamiento y Sociobiología de Abejas, Instituto de Biodiversidad y Biología Experimental y Aplicada (IBBEA), CONICET–Universidad of Buenos Aires, Buenos Aires, Argentina

^3^ Instituto Nacional de Biodiversidad, Quito, Ecuador

^4^ Departamento de Biología, Escuela Politécnica Nacional, Quito, Ecuador

^†^These authors contributed equally to this work

^*^Corresponding Authors:

Raul Ontaneda Gallegos

Ave 12 de Octubre 1076, Quito, 170143, Ecuador

Email address: <raul-fog@outlook.com>

Emilia Moreno C.

Intendente Güiraldes, CABA, Buenos Aires, 1428, Argentina

Email address: [emiliamorenoc@gmail.com](mailto:emiliamorenoc@gmail.com)

This document is a supplement to the main article. It provides detailed observations on the distribution, nesting, floral and non-floral resource use, behavior, and interactions of Xylocopa species in Ecuador, based on museum specimen data, field surveys, and citizen science observations. It also notes on excluded species and morphological remarks that support the checklist decisions made in the main article.

**Results**

***Additional Taxonomic and Ecological Information of Xylocopa Species in Ecuador***

**Subgenus *Neoxylocopa* Michener, 1954**

***Xylocopa* (*Neoxylocopa*) *darwini* Cockerell, 1926**

*Xylocopa darwini* nests in *Bursera graveolens* (palo santo) (MCZ:Ent:815925) and *Cordia lutea* (muyuyo) (MCZ:Ent:815428). Kleptoparasitism by the triangulin larvae of the blister beetle *Cissites maculata* (Swederus, 1787) has been recorded in these nests (MCZ:Ent:810644; MCZ:Ent:816117). Males have been observed engaging in territorial fights (MCZ:Ent:813914). Introduced smooth-billed ani birds (*Crotophaga ani* Linnaeus, 1758) have been recorded preying on this species (iNaturalist: [324733668](https://www.inaturalist.org/observations/324733668)). In the Galápagos, this bird is the only established non-native vertebrate and may exert significant predation pressure on *X. darwini* populations (Cisneros-Heredia 2018).

***Xylocopa* (*Neoxylocopa*) *lachnea* Moure, 1951**

This species is common on the eastern slopes of the Andes and may reach inter-Andean urban areas such as Ambato in the Hoya del Patate via the Pastaza River basin. Specimens have been recorded throughout this corridor, including localities such as Baños de Agua Santa. We also directly observed kleptoparasitism by larvae of *Cissites maculata*. The affected individual (QCAZI280695) appeared lethargic and exhibited erratic flight behavior

***Xylocopa* (*Neoxylocopa*) *similis* Smith, 1874**

One specimen was collected using a light trap (QCAZI280535) during the night. *Xylocopa* species are generally diurnal, although a few nocturnal species are known (Somanathan et al., 2008). This record likely represents occasional attraction to artificial light rather than true nocturnal activity, as citizen science observations show this species active during the day. Artificial lighting could be influencing their behavior. Several female specimens exhibited pronounced wear on the mandibles, likely associated with nesting activity. Some individuals (USNMENT01604802; MECN 12812) differed from typical descriptions, displaying a dark blue integument with reddish accents and sparse, lighter ferruginous pubescence. We considered this variation to be intraspecific, with the cause still unknown. Nevertheless, due to this uncertainty, these specimens were excluded from the dataset.

***Xylocopa* (*Neoxylocopa*) sp.2**

Captured in a cacao field (QCAZI280638), indicating occurrence in human-altered agricultural environments and suggesting that this taxon may persist in disturbed or semi-managed habitats.

**Subgenus *Schonnherria* Lepeletier, 1841**

***Xylocopa* (*Schonnherria*) *ecuadorica,* Cockerell 1909**

Although this species is primarily associated with the Chocó region, it may reach the Inter-Andean valleys around Quito via the Guayllabamba River basin. In the Cumbayá and Pomasqui valleys. There we observed this species nesting in the wood of *Schinus molle* (Anacardiaceae) (QCAZI280753; QCAZI280754). In one nest (Figure 7e), two males were observed sharing the same cavity (iNaturalist: [321892858](https://www.inaturalist.org/observations/321892858); third photo). Male carpenter bees are typically territorial (Barrows, 1983), making this observation noteworthy. This does not appear to be related to emergence, both individuals were observed returning to the cavity, suggesting it was being used as a shared resting or shelter site.

***Xylocopa* (*Schonnherria*) *lucida* Smith, 1874**

Specimen data and field observations indicate that this species is attracted to non-floral resources such as rotten meat and human urine, likely associated with salt collection (QCAZI280540; QCAZI280648; QCAZI280649). This behavior is common among Neotropical bees, including *Trigona*, *Melipona*, and *Centris* (Roubik, 1989).

***Xylocopa* (*Schonnherria*) *ornata* Smith, 1874**

*Xylocopa ornata.* Individuals were observed collecting salts in groups on soil, in association with *X. lucida* (Figure 7f).

***Xylocopa* (*Schonnherria*) *viridis* Smith, 1854**

Museum labels at QCAZI indicate that this species is attracted to Van Someren traps baited with decaying shrimp and to necrophagous traps (QCAZI280545; QCAZI280655). In Santa Clara (Pastaza), we directly observed individuals nesting in a dry log of *Rollinia mucosa* (Annonaceae) (QCAZI280751).

***Xylocopa* (*Schonnherria*) *viridigastra* Lepeletier, 1841**

Several specimens collected at lower altitudes and in coastal areas had been previously identified as *X. viridigastra* but were later confirmed as *X.* (*Neoxylocopa*) sp. 1. These include individuals housed at QCAZI (QCAZI18899, QCAZI18854–18856, QCAZI18907, QCAZI18911, QCAZI18914, QCAZI18929, QCAZI18901) from Catamayo (Loja), Portoviejo (Manabí), Salinas (Santa Elena), and Atacames (Esmeraldas). The specimens lacked original identification labels so the identifier is unknown. All misidentified specimens were females, and no males were found from these localities. Nevertheless, males of *Neoxylocopa* were present in the same collecting events, supporting the misidentification. Additional sampling in coastal and lowland regions is needed to better assess the true distribution of this species in Ecuador.

***Species Excluded Due to Misidentification, Taxonomic or Geographical Uncertainty***

Fourteen species were excluded from this list due to misidentifications or taxonomic issues (Table S6).

***Xylocopa teneralis***

This name was applied to a specimen determined by P. D. Hurd in 1959. It is not currently recognized as a valid species, and the specimen is listed only at the genus level in GBIF (USNMENT01611254).

**Subgenus *Koptortosoma* Gribodo 1894**

***Xylocopa (Koptortosoma) buruana* Lieftinck, 1956**

No determiner is recorded for this specimen (USNMENT01606387). The subgenus *Koptortosoma* is restricted to the Old World (Leys et al., 2002), making any Ecuadorian record biogeographically implausible.

***Xylocopa (Koptortosoma) caffra* (Linnaeus, 1767)**

This species is native to Africa (Mawdsley, 2019). The specimen label (HYMA02896) reads “Nieuwoudt’s Pass – Ecuador”; however, Nieuwoudt’s Pass is located in South Africa, indicating a labeling error.

**Subgenus *Megaxylocopa* Hurd & Moure, 1963**

***Xylocopa (Megaxylocopa) nautlana* Cockerell, 1904**

All the completely black *Megaxylocopa* females in the QCAZI collection were previously labeled as *X. nautlana*. Our examination using the diagnostic key of Mérida-Rivas et al. (2022) confirmed that all these black females are in fact *X. frontalis*. *Xylocopa nautlana* is restricted to Central America and Mexico and does not occur in Ecuador (Moure & Melo, 2023).

**Subgenus *Neoxylocopa* Michener, 1954**

***Xylocopa (Neoxylocopa) brasilianorum* (Linnaeus, 1767)**

Excluded because the original description was based on a male, and the name has since been incorrectly applied to various melanic females of different *Xylocopa* species (Marchi & Alves-dos-Santos, 2013). No confirmed Ecuadorian material assignable to this species was found.

***Xylocopa (Neoxylocopa) bruesi* Cockerell, 1914**

No specimens were available in museum collections or GBIF, and the species has only been reported from Ecuador in the literature (Rasmussen, 2004; Hurd, 1978). The specimen mentioned by Rasmussen could not be located in any national collection. In the same catalog that lists the species as present in Ecuador, Hurd (1978) noted that *X. bruesi*, *X. bariwal* Maidl, 1912 and *X. lachnea* are closely related and may represent a single species. In the entry for *X. lachnea,* he also suggested it could be a synonym of *X. bruesi*. Observations by Ascher cited in Montalva et al. (2013) indicate that Chilean specimens labeled as *X. bruesi* differ from the type of *X. brasilianorum bruesi* at the American Museum of Natural History in labral tubercles, wing color and tergal hairs length, putting in doubt the true range of this species. Examination of photographs of the specimen housed at the MCZ (MCZ: Ent:810841) also revealed no clear morphological differences between *X. bruesi* and the specimens identified as *X. lachnea* in Ecuadorian collections. However, Hurd and Moure (1963) recognized both species as distinct based on male genitalia, and a recent phylogenetic analysis by Melo and Martins (2025) places them in separate clades. A molecular revision of Ecuadorian material currently identified as *X. lachnea* is needed to confirm whether *X. bruesi* is cryptically present in Ecuador. Until then we have decided to not include this species in the catalogue.

***Xylocopa (Neoxylocopa) colona* Lepeletier, 1841**

Ecuadorian records most likely result from misidentification. Hurd (1978) noted that all reported occurrences outside French Guiana were based on erroneous identifications: Galápagos specimens correspond to *X. darwini*; Central American specimens to *Xylocopa* (*Stenoxylocopa*) *strandi* Dusmet & Alonso, 1924; and South American specimens to other taxa. The Moure & Melo catalogue lists *X. colona* only from French Guiana (Moure & Melo, 2023).

***Xylocopa (Neoxylocopa) mastrucata* Pérez, 1901**

*Xylocopa* (*Neoxylocopa*) *mastrucata* Pérez, 1901 is known only from literature sources. Although Moure’s catalog lists it for Ecuador, all subsequent references (Gonzalez et al., 2009; Ospina, 2000) trace back to Hurd (1978), who mentioned its occurrence in Baños and Sabanilla without citing a source or indicating where the specimens are housed. The description by Pérez (1901) notes brown coloration beneath the funicle from the third segment, reddish-brown margins on the ventral segments, coppery to purplish wing reflections, and a dense tuft of long erect hairs around the juxtantennal carina. No specimens showing these features were found in any examined collections, particularly the reddish tones on the sterna. This character could not be directly compared, as there are no available photographs of this body region of the holotype (EY3282) housed at the MNHN uploaded to GBIF. Five field surveys were conducted in Baños at different times of the year and one in Sabanilla, but the species was not encountered. Nevertheless, its presence cannot be entirely ruled out, as it may persist undetected. The reported localities are also biogeographically inconsistent, lying on opposite sides of the Andes with distinct climates and vegetation.

***Xylocopa (Neoxylocopa) rufidorsum* Enderlein, 1913**

Previous records from Ecuador are here considered misidentifications of *X*. *similis*. The former lacks ferruginous pubescence on T1 (Lucia & Gonzalez, 2017). The two specimens labeled as *X. rufidorsum* from Ecuador (SDNHM247348 and SDNHM247349), housed at the San Diego Natural History Museum, were examined through photographs available on GBIF. Both display ferruginous hairs on T1, confirming that they correspond to *X. similis*.

***Xylocopa (Neoxylocopa) sonorina* Smith, 1874**

Synonym: *Xylocopa* (*Neoxylocopa*) *varipuncta* Patton, 1879

One individual identified as this species (MZUTI:Invertebrados:19259). Native to the southwestern United States and Mexico (Mawdsley, 2017b). Although introduced to several Pacific islands and Canada (Sheffield et al., 2020), there is no evidence of its introduction into Ecuador.

***Xylocopa (Neoxylocopa) rotundiceps* Smith, 1874**

A single specimen labeled as this species (USNMENT01615808) was located, but *X. rotundiceps* is otherwise known only from Brazil (Moure & Melo, 2023; Lucia et al., 2014). The available photograph does not allow reliable confirmation. This specimen was originally listed by Cockerell (1914) among *X. transitoria* females from Guayaquil and appears to have been reidentified at an unknown date by an unspecified individual at the MCZ.

***Xylocopa (Neoxylocopa) transitoria* Pérez, 1901**

Cockerell (1914) reported four females of *X*. *transitoria* collected by C. Brues in Guayaquil, but only two remain identified as such (USNMENT01124163; USNMENT01614982). USNMENT01615808 is one of the specimens listed by Cockerell and appears to have been reidentified as *X. rotundiceps* at an unknown date. The determination is attributed to the Museum of Comparative Zoology (MCZ), though the individual responsible is not specified. Another specimen from the same series, USNMENT01611158, is now identified only at genus level.

**Subgenus *Schonnherria* Lepeletier, 1841**

***Xylocopa* (*Schonnherria*) *varians* Smith, 1874**

Specimens previously identified as *X. varians* were personally examined in the QCAZI collection and reclassified as *X. ecuadorica* and *X. lucida* based on original descriptions and coloration (Cockerell, 1909, 1912; Villamizar et al., 2020). One individual of *Xylocopa* sp. 3 was also identified as *X. varians.*

***Xylocopa* sp. aff. *ecuadorica/viridis***

Examination of Amazonian material revealed specimens with intermediate morphological characteristics between *X. ecuadorica* and *X. viridis* (Fig. 5b). These specimens do not fully match the coloration patterns of either species. They differ from *X. ecuadorica* by having pale setae on the foretarsus and tibia and a reddish to brownish-red pubescence on the mesosoma. Both males and females exhibiting this intermediate coloration were observed. Specimens showing this morphology are treated as an informal group and are labeled as *Xylocopa* sp. aff. *ecuadorica/viridis* (hereafter *Xylocopa* sp. aff. ecua–vir) in maps and molecular analyses. A conservative taxonomic approach was adopted for the checklist. Specimens were assigned to either species only when their morphology closely matched original descriptions and diagnostic characters in available keys, with nesting data gathered in field from both species to associate males and females. Specimens that could not be confidently assigned were excluded from the checklist and treated as this intermediate morphotype.

**DNA barcode.** Specimens identified as *X. ecuadorica* from Ecuador and Costa Rica, the intermediate morphotypes from Ecuador and Peru (Madre de Dios), and *X. viridis* from Ecuador share the same BIN (AET4851) (Table S4).

**References**

Barrows, E. M. (1983). Male territoriality in the carpenter bee *Xylocopa virginica*. *Animal Behaviour, 31*(3), 806–813. <https://doi.org/10.1016/S0003-3472(83)80237-1>

Cisneros-Heredia, D. F. (2018). The hitchhiker wave: Non-native small terrestrial vertebrates in the Galapagos. In M. Torres & C. Mena (Eds.), *Understanding invasive species in the Galapagos Islands: Social and ecological interactions in the Galapagos Islands*. Springer, Cham. <https://doi.org/10.1007/978-3-319-67177-2_7>

Cockerell, T. D. A. (1909). Descriptions and records of bees. XXIII. *Annals and Magazine of Natural History, 8*(4), 393–504.

Cockerell, T. D. A. (1912). Descriptions and records of bees. XLVII. *Annals and Magazine of Natural History, 8*(10), 484–493.

Cockerell, T. D. A. (1914). Bees from Ecuador and Peru. *Journal of the New York Entomological Society, 22*, 306–328.

Gonzalez, V. H., Gonzalez, M. M., & Cuellar, Y. (2009). Notas biológicas y taxonómicas sobre los abejorros del maracuyá del género *Xylocopa* (Hymenoptera: Apidae, Xylocopini) en Colombia. *Acta Biológica Colombiana, 14*(2), 31–40.

Hurd, P. D. (1978). *An annotated catalog of the carpenter bees (genus Xylocopa Latreille) of the Western Hemisphere (Hymenoptera: Anthophoridae)*. Smithsonian Institution Press.

Hurd, P. D., & Moure, J. S. (1963). *A classification of the large carpenter bees (Xylocopini) (Hymenoptera: Apoidea)*. University of California Press.

Lucia, M., Alvarez, L. J., & Abrahamovich, A. H. (2014). Large carpenter bees in Argentina: Systematics and notes on the biology of *Xylocopa* subgenus *Neoxylocopa* (Hymenoptera: Apidae). *Zootaxa, 3754*(3). <https://doi.org/10.11646/zootaxa.3754.3.1>

Lucia, M., & Gonzalez, V. H. (2017). New species and designation of primary types in Neotropical carpenter bees of the genus *Xylocopa* Latreille (Hymenoptera, Apidae). *Journal of Hymenoptera Research, 61*, 31–48. <https://doi.org/10.3897/jhr.61.20345>

Marchi, P., & Alves-dos-Santos, I. (2013). As abelhas do gênero *Xylocopa* Latreille (Xylocopini, Apidae) do Estado de São Paulo, Brasil. *Biota Neotropica, 13*(2), 249–269. <https://doi.org/10.1590/S1676-06032013000200025>

Mawdsley, J. R. (2017). *Large carpenter bees: A guide to species of Xylocopa (Neoxylocopa) from North and Central America*. Pineway Press.

Mawdsley, J. R. (2019). Cladistic analysis of the *Xylocopa caffra* (Linnaeus, 1767) species-group (Insecta: Hymenoptera: Apidae). *Tropical Zoology, 32*(1), 49–57. <https://doi.org/10.1080/03946975.2018.1549866>

Melo, G. A. R., & Martins, A. C. (2025). The large carpenter bees (*Xylocopa*, Apidae) of the Neotropical region: Phylogenetics, classification and biogeography. *Zoologica Scripta, 54*(6), 884–896. <https://doi.org/10.1111/zsc.70015>

Mérida-Rivas, J. A., Hinojosa-Díaz, I. A., Ayala-Barajas, R., Barrientos-Villalobos, S., Pozo, C., & Vandame, R. (2022). Revision of carpenter bees of the subgenus *Neoxylocopa* Michener (Hymenoptera: Apidae) from Mexico and Mesoamerica. *Zootaxa, 5158*(1), 1–67. <https://doi.org/10.11646/zootaxa.5158.1.1>

Montalva, J. M., Allendes, J. L., & Lucia, M. (2013). The large carpenter bee *Xylocopa augusti* (Hymenoptera: Apidae): New record for Chile. *Journal of Melittology, 12*. <https://doi.org/10.17161/jom.v0i12.4480>

Moure, J. S., & Melo, G. A. R. (2023). Xylocopini Latreille, 1802. In J. S. Moure, D. Urban, & G. A. R. Melo (Eds.), *Catalogue of bees (Hymenoptera, Apoidea) in the Neotropical Region – online version*. <https://www.moure.cria.org.br/catalogue>

Leys, R., Cooper, S. J. B., & Schwarz, M. P. (2002). Molecular phylogeny and historical biogeography of the large carpenter bees, genus *Xylocopa* (Hymenoptera: Apidae). *Biological Journal of the Linnean Society*, *77*(2), 249–266. [https://doi.org/10.1046/j.1095-8312.2002.00108.x](https://doi.org/10.1046/j.1095-8312.2002.00108.x%20)

Ospina, M. (2000). *Abejas carpinteras (Hymenoptera: Apidae: Xylocopinae: Xylocopini) de la región Neotropical*. Biota Colombiana.

Pérez, J. (1901). Contribution à l’étude des Xylocopes. *Actes de La Société Linnéenne de Bordeaux, 56*, 1–128.

Rasmussen, C. (2004). Bees from Southern Ecuador. *Lyonia, 7*, 29–35.

Roubik, D. W. (Ed.). (1989). *Ecology and natural history of tropical bees* (1st ed.). Cambridge University Press. <https://doi.org/10.1017/CBO9780511574641>

Sheffield, C., Heron, J., & Musetti, L. (2020). *Xylocopa sonorina* Smith, 1874 from Vancouver, British Columbia, Canada (Hymenoptera: Apidae, Xylocopinae) with comments on its taxonomy. *Biodiversity Data Journal, 8*, e49918. <https://doi.org/10.3897/BDJ.8.e49918>

Somanathan, H., Borges, R. M., Warrant, E. J., & Kelber, A. (2008). Visual ecology of Indian carpenter bees I: Light intensities and flight activity. *Journal of Comparative Physiology A, 194*(1), 97–107. <https://doi.org/10.1007/s00359-007-0291-1>

Villamizar, G., Fernández, F., & Vivallo, F. (2020). Synopsis of the carpenter bee subgenus *Xylocopa* (*Schonnherria*) Lepeletier, 1841 (Hymenoptera: Apidae) in Colombia, with designation of lectotypes and the description of two new species. *Zootaxa, 4789*(2). <https://doi.org/10.11646/zootaxa.4789.2.1>
